# Establishment and translational evaluation of animal models for skin wound healing: a systematic review

**DOI:** 10.3389/fphys.2026.1800001

**Published:** 2026-04-16

**Authors:** Ying Zhang, Kai Leng, Miao Dong, Jinxuan Wu, Zi Wang, Qiao Zhu, Xinghua Gao, Yan Sun

**Affiliations:** 1 Department of Dermatology, The First Hospital of China Medical University, Shenyang, China; 2 National Health Commission (NHC) Key Laboratory of Immunodermatology, Ministry of Education Key Laboratory of Immunodermatology, National Joint Engineering Research Center for Diagnosis and Treatment of Immunologic Skin Diseases, The First Hospital of China Medical University, Shenyang, China; 3 Department of Thoracic Surgery, The First Hospital of China Medical University, Shenyang, China

**Keywords:** animal models, diabetic, review, skin wound healing, translational research

## Abstract

**Background:**

Skin wounds, encompassing acute injuries and chronic refractory ulcers, impose substantial physical and economic burdens globally. While animal models are indispensable for dissecting wound healing pathophysiology and testing therapeutic interventions, the discordance between preclinical findings and clinical outcomes remains a critical challenge.

**Methods:**

To provide a standardized reference for model selection, we conducted a systematic review in accordance with PRISMA guidelines. We comprehensively searched PubMed, Web of Science, and Scopus for studies published between January 1, 2015, and December 31, 2025. Inclusion criteria focused on *in vivo* cutaneous wound models in mice, rats, and rabbits that reported quantitative outcomes (e.g., closure kinetics, histology, molecular markers). Studies lacking separate control groups or sufficient methodological detail were excluded. The methodological quality of included studies was assessed using SYRCLE’s risk of bias tool.

**Results:**

A total of 129 studies met the inclusion criteria and were synthesized. We systematically categorized and evaluated mainstream models: (1) Acute wounds: Rodent incisional/excisional models facilitate high-throughput screening but are limited by contraction-dominant healing, whereas rabbit ear models better approximate human re-epithelialization. (2) Chronic wounds: Streptozotocin (STZ)-induced diabetic models in mice and rats predominate but often lack the macrovascular complications of human ulcers, necessitating novel composite models incorporating ischemia and biofilm infection. (3) Pathological scarring: Tension-induced models (e.g., suture anchoring, mechanical stretching) are identified as critical for studying mechanotransduction pathways (e.g., YAP/TAZ) absent in traditional unstressed models. Furthermore, our review identifies a pervasive male bias in study design. We highlight that sex steroids critically modulate inflammation and angiogenesis—with estrogen typically promoting and androgens delaying repair—necessitating the inclusion of both sexes or specific hormone-depleted models (e.g., ovariectomized females) to improve clinical predictive value.

**Conclusion:**

No single animal model perfectly recapitulates human cutaneous repair. Based on the synthesis of 129 studies, we propose a hierarchical translational framework: utilizing genetically tractable mice for mechanistic discovery, rats for longitudinal pharmacological screening, and rabbits or porcine models for the validation of scar quality and epithelial closure prior to clinical trials.

## Highlights

- Systematically summarizes the mainstream animal models in skin wound healing research, including acute wound (incisional, excisional, thermal), chronic diabetic wound, and tension-induced scar models, focusing on the establishment methods and standardized procedures of three core species: mice, rats, and rabbits.
- Clarifies the core value of models from different species: mouse models are cost-effective with strong genetic manipulability, suitable for mechanism exploration; rat models facilitate repeated sampling, adapting to longitudinal observation and medium-scale validation; rabbit models have skin structure close to humans, showing prominent translational value in studies on scar formation and epithelial repair.
- Emphasizes the core logic of model selection—based on the dominant healing mechanism (contraction vs. re-epithelialization) and research endpoints, and proposes a hierarchical research approach of “mechanism discovery in mice → validation in rats → scar phenotype confirmation in rabbits”.
- Covers the latest progress in model optimization, including splinting technology to reduce contraction interference, diabetic models with multiple comorbidities (e.g., ZDSD rats), wireless tension monitoring devices, etc., improving the accuracy of clinical translation.
- Provides standardized model selection references for mechanism research, drug screening, and clinical translation of skin wound healing, helping bridge the gap between basic research and clinical application.

## 1 Introduction

Skin wounds are defined as injuries or disruptions to the skin’s normal architecture caused by trauma, laceration, incision, or bruising (Nourian Dehkordi et al., 2019). Wound healing is a complex, highly orchestrated biological process encompassing the sequential phases of hemostasis, inflammation, proliferation, and tissue remodeling (Tottoli et al., 2020). Clinically, the classification of wounds as acute or chronic—based on pathophysiological features and healing outcomes—serves as a foundational framework for both research design and clinical practice (Tottoli et al., 2020). Acute wounds typically stem from insults such as surgical incisions, excisions, or thermal injury. For these wounds, repair involves a dynamic cascade of cellular signaling and behavioral events that ensure rapid skin barrier closure (Werner and Grose, 2003). High redundancy and compensatory mechanisms within this cascade mean minor perturbations rarely impair healing; however, sufficient systemic disruption can lead to abnormalities or healing failure. In contrast to the robust repair seen in acute injuries, chronic wounds—defined as unhealed wounds lasting ≥12 weeks—represent a failure of this orchestrated process (Guest et al., 2015; Lindholm and Searle, 2016). These wounds predominantly affect the elderly and diabetic populations, posing significant clinical challenges. Among these, diabetic wounds are the most heavily studied. Persistent hyperglycemia directly induces healing defects by impairing hematopoietic cell function (Stegenga et al., 2008), triggering cellular senescence (Short-term sustained hyperglycaemia fosters an archetypal senescence-associated secretory phenotype in endothelial cells and macrophages, 2018), and leading to the formation of advanced glycation end products (AGEs) (Gkogkolou and Böhm, 2012). These molecular disruptions induce inflammation and reactive oxygen species (ROS), inhibit neovascularization via HIF-1α downregulation (Thangarajah et al., 2009), and result in long-term microvascular damage (Walsh et al., 2016). Consequently, Diabetic Foot Ulcers (DFUs) represent the most prevalent refractory skin wounds. Approximately 15–20% of DFU infections require amputation (Armstrong et al., 2017; Petersen et al., 2022), and the five-year mortality rate for DFU patients is nearly 30% (Skrepnek et al., 2015; Lipsky et al., 2020; Petersen et al., 2022). In the United States alone, the direct annual costs associated with DFUs are projected to reach $9–13 billion (Armstrong et al., 2023), highlighting the urgent need for effective therapeutic strategies.

While metabolic disorders typified by diabetes are a primary driver of chronic wounds, clinical practice encounters a diverse spectrum of refractory wounds arising from distinct etiologies beyond diabetes. Factors such as ischemia, autoimmune dysregulation, and radiation exposure present unique pathophysiological mechanisms that must be accurately reflected in preclinical modeling.

### 1.1 Pressure injuries

Pressure injuries, historically termed pressure ulcers or decubitus ulcers, result from localized damage to the skin and underlying soft tissue due to prolonged, unrelieved pressure or pressure in combination with shear (Al-Nadaf et al., 2024). The central pathophysiological event involves sustained mechanical loading that exceeds capillary perfusion pressure, precipitating local tissue ischemia and hypoxia. At the cellular level, mechanical deformation induces cytoskeletal disruption and ion channel dysfunction, triggering apoptosis (Yin et al., 2018). Subsequent ischemia-reperfusion injury generates a surge of reactive oxygen species (ROS), exacerbating inflammation and tissue necrosis (Green synthesis of silver nanoparticles from aqueous extract of scutellaria barbata and coating on the cotton fabric for antimicrobial applications and wound healing activity in fibroblast cells (L929), 2021). Furthermore, lymphatic dysfunction leads to the accumulation of interstitial fluid and metabolic waste, creating a toxic microenvironment that arrests repair (Sullivan et al., 2001). Preclinical models typically simulate this by applying external pressure to the dorsal skin of animals (e.g., mice, rats) using magnetic plates or static weights to induce controlled ischemia and tissue damage, thereby providing a platform to evaluate novel therapeutic strategies (Green synthesis of silver nanoparticles from aqueous extract of scutellaria barbata and coating on the cotton fabric for antimicrobial applications and wound healing activity in fibroblast cells (L929), 2021).

### 1.2 Ischemic wounds (peripheral arterial disease)

Ischemic wounds driven by peripheral arterial disease (PAD) are caused by atherosclerosis, which occludes arteries and drastically restricts blood flow to the extremities (Campia et al., 2019). This chronic state of hypoxia and nutrient deprivation impairs cellular metabolism, inhibits fibroblast proliferation, and suppresses angiogenesis (Everett and Mathioudakis, 2018). Furthermore, the lack of immune cell recruitment increases infection susceptibility. In preclinical research, this pathology is typically modeled by surgically ligating or excising the femoral arteries in mice or rabbits, creating a controlled environment to study healing dynamics under ischemic stress and evaluate pro-angiogenic therapies (Mohamad Yusoff et al., 2021).

### 1.3 Autoimmune ulcers

Ulcers associated with autoimmune diseases, such as systemic lupus erythematosus (SLE) or vasculitis, result from immune system dysregulation. Autoantibodies and immune complexes often target endothelial cells, triggering necrotizing vasculitis and downstream ischemic necrosis (Shanmugam et al., 2017). A persistent inflammatory state, driven by elevated cytokines like TNF-α, degrades tissue architecture and blocks reparative mechanisms (Nor et al., 2023). Modeling these wounds is complex and relies heavily on genetically engineered animals (e.g., MRL/lpr mice) that spontaneously develop autoimmune lesions, allowing researchers to study wound impairment in the context of systemic immune dysfunction (Immune-competent human skin disease models, 2016).

### 1.4 Tumor-related and post-radiation wounds

Tumors may cause direct ulceration, but severe skin damage frequently arises from radiation therapy. Ionizing radiation causes DNA double-strand breaks, depleting stem cells and fibroblasts essential for the skin barrier (Cui et al., 2024). Late-stage effects include progressive fibrosis and microvascular occlusion, creating a hypoxic, sclerotic environment prone to refractory ulcers (Sorg et al., 2017). Preclinical models simulate this by subjecting animals to high-dose irradiation prior to wounding, mimicking the delayed healing and fibrosis seen in clinical radiation injuries (Cui et al., 2024).

### 1.5 Biochemical dysregulation and research gaps

Regardless of etiology, these chronic wounds share a dysregulated biochemical profile characterized by a sustained imbalance of pro-inflammatory mediators (e.g., IL-1, IL-6, TNF-α), matrix metalloproteinases (MMPs), and growth factors (Whittam et al., 2016). While these markers are theoretically critical for differentiation and treatment, practical application lags behind. Current experimental models often prioritize visual closure rates over rigorous analysis of histological quality or functional recovery, failing to fully capture the complex molecular landscape of chronic wounds (Nunan et al., 2014).

To bridge this gap, animal models serve as irreplaceable platforms for dissecting wound healing pathophysiology. However, their clinical predictive value is limited by inherent physiological constraints. A primary limitation is the physiological difference between species. Rodent skin possesses the panniculus carnosus muscle, which allows wounds to heal primarily via contraction (Zomer and Trentin, 2018). In contrast, human skin heals through re-epithelialization and granulation tissue formation. This fundamental difference can lead to misleading interpretations when translating rodent data to human therapies (Abdullahi et al., 2014). Furthermore, while porcine (Sus scrofa domesticus) skin is structurally and physiologically the closest to human skin (Sullivan et al., 2001), it remains underutilized due to high costs and logistical challenges (Seaton et al., 2015). Crucially, sex-based differences in wound healing—driven by the opposing effects of estrogen (anti-inflammatory, pro-angiogenic) and androgen (pro-inflammatory)—are increasingly recognized as determinants of outcomes (Larouche et al., 2018). Yet, a predominance of male animals is used in preclinical studies to avoid estrous cycle variability, creating a significant translational gap for female patient populations. Finally, inadequate simulation of the chronic disease microenvironment (e.g., polymicrobial infection, age-related variability) and a lack of rigorous quality appraisal (randomization, blinding) in many studies further hinder translation (Thomason et al., 2015).

However, a critical methodological pitfall persists in current preclinical research: the over-reliance on macroscopic wound closure rate as the primary surrogate for healing efficacy. In rodents, the panniculus carnosus muscle drives rapid wound contraction, a mechanism that can account for up to 80–90% of wound area reduction but is largely absent in human skin repair, which relies on re-epithelialization and granulation tissue formation (Fischer et al., 2023). Consequently, interventions that merely accelerate contraction in mice may fail to translate to human patients, potentially leading to “false positive” outcomes. By synthesizing a large body of literature, this review systematically summarizes the establishment methods of mainstream animal wound models—including full-thickness excision, burn, incision, and diabetic wounds. We critically evaluate the discordance between contraction-based and epithelialization-based healing and address the limitations of current approaches. Finally, we propose innovative strategies for future model design and optimization, emphasizing the need for better reporting standards and model selection based on specific clinical indications. This review aims to offer a reliable, practical resource for investigators, facilitating advancements in the understanding and treatment of skin wound healing.

### 1.6 Acute skin wounds

Acute skin wounds typically result from surgical incisions or clean lacerations, supporting the regeneration of connective tissue and epithelium without fibrosis formation (Velnar et al., 2009; Alhajj and Goyal, 2024). These wounds heal completely within 2–3 weeks, although in small animal models like mice and rats, the timeline is often accelerated due to their higher metabolic rates and distinct healing mechanisms.

### 1.7 Wound healing process

Acute wound healing involves a sequence of coordinated molecular and cellular events, ultimately restoring normal tissue structure. This process unfolds across four interconnected phases: hemostasis (bleeding cessation), inflammation (immune response activation), proliferation (new tissue growth), and remodeling (tissue refinement and strengthening) (Larouche et al., 2018). Understanding these phases is critical for selecting appropriate time points for data collection in animal studies, ensuring that experimental endpoints align with the specific biological events of interest.

#### 1.7.1 Hemostasis stage

Immediately following wounding, vascular constriction and fibrin clot formation are rapidly initiated to achieve hemostasis (Guo and DiPietro, 2010). The clot and surrounding wound tissue release various growth factors and pro-inflammatory cytokines, including fibroblast growth factor (FGF), platelet-derived growth factor (PDGF), transforming growth factor (TGF)-β, and epidermal growth factor (EGF) (Guo and DiPietro, 2010). This provisional fibrin matrix not only stops bleeding but also serves as a scaffold for infiltrating cells, while the released granules from degranulating platelets provide the initial chemotactic gradient necessary to recruit neutrophils and monocytes from the circulation.

#### 1.7.2 Inflammation stage

Skin damage triggers a complex immune response to eliminate pathogens invading the wound site. Damage-induced signals activate both pathogen-associated molecular patterns (PAMPs) from bacterial components and damage-associated molecular patterns (DAMPs) from necrotic cells and injured tissues (Wilkinson and Hardman, 2020). By binding to pattern recognition receptors, PAMPs and DAMPs initiate downstream inflammatory pathways, activating immune cells such as T cells, macrophages, mast cells, and Langerhans cells (Chen and DiPietro, 2017).

Neutrophils are recruited to the wound site via cytokines including lipopolysaccharide (LPS), bacterial endotoxins, interleukin-1 (IL-1), and tumor necrosis factor-α (TNF-α) (Martin and Leibovich, 2005). Once pro-inflammatory signaling pathways (e.g., NF-κB) are activated, neutrophils and other wound-resident cells secrete additional cytokines (Kolaczkowska and Kubes, 2013). Following phagocytosis of pathogens and necrotic tissue, neutrophils release reactive oxygen species (ROS), antimicrobial peptides, arachidonic acids, and proteolytic enzymes (Segel et al., 2011). Notably, they also utilize extracellular traps—DNA-based filamentous structures embedded with antimicrobial peptides and cytotoxic histones—to capture and eliminate pathogens (Brinkmann et al., 2004).

Monocytes differentiate into macrophages upon entering the wound microenvironment. As key effector cells in tissue restoration, macrophages exhibit considerable phenotypic plasticity (Das et al., 2015). Activated by pro-inflammatory stimuli such as interferon-γ (IFN-γ) and LPS, these cells mediate inflammation by secreting growth factors (e.g., PDGF, vascular endothelial growth factor [VEGF]) and pro-inflammatory cytokines (e.g., IL-1, IL-6, TNF-α). This initial population is often referred to as the M1-polarized (pro-inflammatory) phenotype. By phagocytosing apoptotic neutrophils, macrophages become the primary inflammatory mediators (Delavary et al., 2011). Subsequently, the ingestion of apoptotic cells promotes a phenotypic switch from M1 to M2 (anti-inflammatory/pro-reparative) macrophages. M2 macrophages are crucial for the resolution of inflammation, secreting IL-10 and TGF-β to suppress immune responses and promote tissue repair. The synergistic actions of macrophages and neutrophils clear debris, pathogens, and pro-inflammatory cells while promoting tissue repair and efficient wound resolution. Additionally, anti-inflammatory regulatory T cells have been shown to facilitate tissue healing in murine models (Nosbaum et al., 2016), and mast cells contribute to early inflammation by releasing histamine, which enhances neutrophil recruitment (Weller et al., 2006).

#### 1.7.3 Proliferative stage

This phase is characterized by the robust activation of multiple cell types—including keratinocytes, fibroblasts, macrophages, and endothelial cells—which collectively regulate wound closure, extracellular matrix (ECM) deposition, and angiogenesis. Within the first 12 hours post-injury, keratinocytes are activated by diverse stimuli: mechanical tension, electrical gradients, hydrogen peroxide, pathogens, growth factors, and cytokines (Shaw and Martin, 2016). Under these cues, keratinocytes at the wound periphery undergo a partial epithelial-mesenchymal transition (EMT), acquiring migratory and invasive properties (Li et al., 2007).

In the nascent epidermis, keratinocytes secrete matrix metalloproteinases (MMPs) to facilitate migration and deposit ECM proteins, thereby reconstituting the basement membrane (Rousselle et al., 2019). Fibroblasts are critical for transforming the fibrin-rich provisional matrix into structurally stable granulation tissue. Resident and mesenchymal-derived fibroblasts respond to molecular signals (e.g., TGF-β, PDGF) from platelets, endothelial cells, and macrophages, adopting a pro-fibrotic phenotype, secreting ECM proteins, or differentiating into myofibroblasts—all essential for wound contraction (Li et al., 2007). In rodent models, the differentiation of fibroblasts into alpha-smooth muscle actin (α-SMA) positive myofibroblasts is particularly pronounced, driving the rapid contraction of the panniculus carnosus muscle, a mechanism less dominant in human wound healing.

Angiogenesis, the formation of new blood vessels, meets the metabolic demands of rapidly proliferating healing tissue. Hypoxia induces angiogenesis by upregulating hypoxia-inducible factor (HIF) and cyclooxygenase-2 (COX-2), which in turn promote the release of VEGF and other pro-angiogenic factors (Huang et al., 2005). VEGF inhibits endothelial cell apoptosis by upregulating anti-apoptotic proteins such as BCL-2 (Cai et al., 2003), while the fibrin matrix supports angiogenesis by facilitating endothelial cell migration (Kalebic et al., 1983). Pericytes are then recruited to stabilize these nascent vessels, ensuring functional perfusion.

#### 1.7.4 Remodeling stage

Fibroblasts are the primary cells responsible for ECM remodeling: they replace early fibrin clots with hyaluronic acid, fibronectin, and proteoglycans, and synthesize mature collagen fibers in the late stages of wound healing (Darby et al., 2014). As healing progresses, type I collagen gradually replaces type III collagen, increasing scar tensile strength (Park et al., 2015). This ratio of Type I to Type III collagen is a standard histological metric in animal studies for evaluating the maturity and strength of the healed tissue. Elastin—a key component of the dermal ECM—must assemble into elastic fibers to maintain skin elasticity. This remodeling process is tightly regulated by the balance between MMPs (which degrade matrix) and their endogenous inhibitors, the tissue inhibitors of metalloproteinases (TIMPs). An imbalance in the MMP/TIMP ratio can lead to pathological outcomes such as hypertrophic scarring or chronic non-healing wounds. Eventually, endothelial cells, fibroblasts, and macrophages either migrate away from the injury site or undergo apoptosis, the wound healing response subsides, and scar formation ensues (Larouche et al., 2018). In successful acute healing, the final tissue possesses approximately 80% of the tensile strength of uninjured skin, a functional recovery endpoint frequently measured in incision models.

## 2 Methods

### 2.1 Protocol and registration

This systematic review was conducted in strict accordance with the Preferred Reporting Items for Systematic Reviews and Meta-Analyses (PRISMA) guidelines (Parums, 2021). The review protocol was defined *a priori* to ensure transparency.

### 2.2 Data sources and search strategy

We performed a comprehensive literature search across three major databases: PubMed, Web of Science, and Scopus. The search timeframe spanned from January 1, 2015, to December 31, 2025. The final search update was conducted on December 31, 2025, to ensure the inclusion of the most recent evidence.

To address the requirement for reproducibility and minimize selection bias (Reviewer Comment 7), specific search strategies were constructed using a combination of Medical Subject Headings (MeSH) and free-text keywords connected by Boolean operators (AND/OR). The search syntax was structured into three logical blocks:

1. Animal models: (“Models, Animal”[Mesh] OR “Animal Experimentation” OR “Mice”[Mesh] OR mouse OR mice OR “Rats”[Mesh] OR rat OR “Rabbits”[Mesh] OR rabbit).
2. Wound pathology: AND (“Skin”[Mesh] OR “Wound Healing”[Mesh] OR skin wound OR incisional wound OR excisional wound OR burn wound OR diabetic wound OR chronic wound OR hypertrophic scar OR fibrosis).
3. Methodological constraints: AND (splint OR ring OR silicone frame OR stretch OR tension OR wound creation OR perioperative management OR quantitative outcome).

The exact search strings were adapted to the specific syntax rules of each database. The complete search history and detailed search strings for all databases are provided in **Supplementary Table 1**.

### 2.3 Eligibility criteria

Studies were selected based on the following PICOS (Population, Intervention, Comparison, Outcome, Study design) criteria:

#### 2.3.1 Inclusion criteria

1. Population: *In vivo* cutaneous wound models in mice, rats, rabbits.
2. Intervention: Detailed descriptions of wound creation (site, size, method), perioperative management (anesthesia, analgesia, antibiotics), and diabetes induction (if applicable).
3. Outcome: Studies reporting quantitative outcomes, including closure kinetics (healing rate, time to closure), histology (epithelial thickness, collagen organization), molecular markers (cytokines, growth factors, α-SMA), tensile strength, or biomechanical properties.
4. Study design: Original full-text articles published in English.

#### 2.3.2 Exclusion criteria

1. Non-cutaneous injury models (e.g., mucosal wounds, internal organ wounds) or *in vitro*/ex vivo studies.
2. Studies with high risk of reporting bias, specifically those lacking sufficient methodological detail to enable replication (e.g., unreported wound size or anesthesia) or missing quantitative data.
3. Reviews, meta-analyses, case reports, and conference abstracts.
4. Studies lacking a separate control group or failing to report sample sizes (n).

### 2.4 Study selection and data extraction

#### 2.4.1 To ensure a systematic selection process

1. Deduplication: All retrieved records were imported into EndNote X9, and duplicates were removed electronically followed by a manual check.
2. Screening: Two independent reviewers (Initials A.A. and B.B.) screened titles and abstracts to exclude irrelevant studies.
3. Full-text review: Potentially eligible articles were retrieved and assessed against the inclusion/exclusion criteria.
4. Resolution of discrepancies: Any disagreements regarding study eligibility were resolved through discussion or by consultation with a third senior reviewer (Initials C.C.) to reach a consensus.

### 2.5 Quality assessment and risk of bias

To evaluate the internal validity of the included studies and address potential methodological flaws, two reviewers independently assessed the risk of bias using the SYRCLE’s Risk of Bias tool tailored for animal studies (Hooijmans et al., 2014). We evaluated ten domains, including:

1. Selection bias: Sequence generation (randomization) and baseline characteristics.
2. Performance bias: Allocation concealment and random housing.
3. Detection bias: Blinding of outcome assessors.
4. Attrition and reporting bias: Incomplete outcome data and selective outcome reporting. Studies were categorized as having a “low,” “high,” or “unclear” risk of bias for each domain. This assessment was critical to determine the reliability of the evidence and its translational value.

### 2.6 Data synthesis and stratification strategy

Given the biological heterogeneity between species, data synthesis was stratified based on the dominant wound closure mechanism (Gilliver, 2010). We acknowledged that rodents primarily heal via panniculus carnosus-driven contraction, whereas humans rely on re-epithelialization. Therefore, to prevent the overinterpretation of contraction kinetics, the analysis framework prioritized:

1. Segregation of data from splinted vs. non-splinted models.
2. Emphasis on histological quality and collagen organization over simple closure rates in loose-skin models.
3. Stratification by anatomical site (e.g., rabbit ear vs. dorsal skin). This mechanism-based framework ensured that the synthesis focused on outcomes with higher external validity and clinical relevance.

### 2.7 Evidence stratification and critical appraisal

To address the heterogeneity in experimental rigor, we stratified the included studies based on the physiological relevance of their outcome measures. Studies were categorized into three tiers of translational evidence: Tier 1 (High Relevance): Studies utilizing splinted rodent models, rabbit ear models, or porcine models that reported histological quality of re-epithelialization and scar architecture (e.g., collagen organization, scar elevation index); Tier 2 (Moderate Relevance): Studies using non-splinted rodent models but incorporating quantitative molecular validation of key human biomarkers (e.g., MMP-9/TIMP-1 ratios, VEGF levels); and Tier 3 (Low Relevance): Studies relying solely on macroscopic wound closure rates in loose-skin animals without histological validation. This stratification allows for a critical evaluation of which models merely achieve “closure” versus those that authentically recapitulate human tissue repair mechanisms.

**Figure 1:**
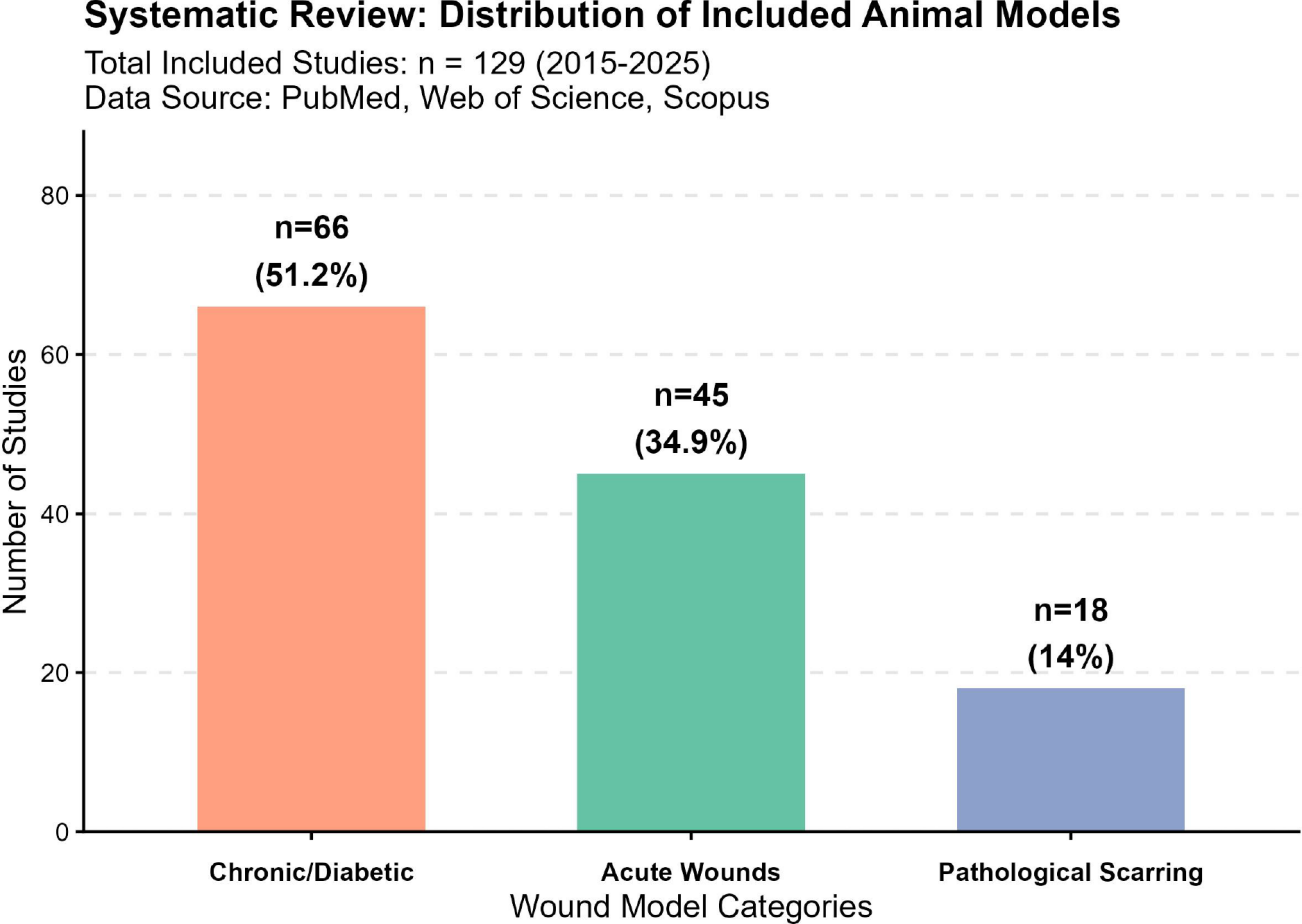
Distribution of animal wound models in current literature (2015–2025).

While the spectrum of chronic wounds in clinical practice encompasses pressure injuries, venous/arterial ischemic ulcers, and autoimmune ulcers, this systematic review primarily stratifies data into Diabetic Wound Models and Tension-Induced Scar Models. This focused selection is driven by three key factors derived from our systematic analysis:

1. Predominance of evidence: Our quantitative analysis of studies (2015–2025) reveals that diabetic wound models constitute the majority (51%, n=75) of preclinical chronic wound research (**Figure 1**). This reflects the urgent global burden of diabetic foot ulcers (DFUs), which have a five-year mortality rate of approximately 30% and annual costs exceeding $9 billion in the US alone. The abundance of high-quality data in this domain allows for a robust, statistically meaningful cross-species comparison that is currently not feasible for less standardized models like pressure ulcers. 2. Mechanistic Representativeness: Diabetic models (particularly STZ-induced and db/dbstrains) effectively recapitulate the core cellular hallmarks of “chronicity”—namely, sustained inflammation (M1 macrophage persistence), impaired angiogenesis, and oxidative stress. These mechanisms serve as a foundational proxy for other non-healing etiologies.
2. Targeted mechanotransduction: We specifically included tension-induced models (e.g., suture anchoring and mechanical stretching) because they address a unique pathophysiological axis: mechanotransduction. Recent studies (2020–2024) have identified mechanical tension as a critical driver of pathological scarring via the YAP/TAZ and Piezo1 pathways. Unlike ischemic or pressure models, tension models provide a distinct platform for evaluating anti-fibrotic therapies, a rapidly evolving field that requires specialized establishment criteria distinct from general ulcer models.

Therefore, to ensure the depth of this review and the rigor of the translational recommendations, we prioritized these two dominant and methodologically distinct categories.

The process began with a comprehensive Database Search, followed by a primary Title and Abstract screening to exclude literature that did not meet the initial criteria. Potentially relevant articles underwent a Full-Text Assessment, where studies with conflicting viewpoints were discussed among reviewers to reach a consensus. The final Included Studies were analyzed to classify the various animal models, summarize their mechanisms, compare their core advantages and disadvantages, and evaluate their translational value and specific application fields in wound healing research.

## 3 Results

### 3.1 Study selection

**Figure 2:**
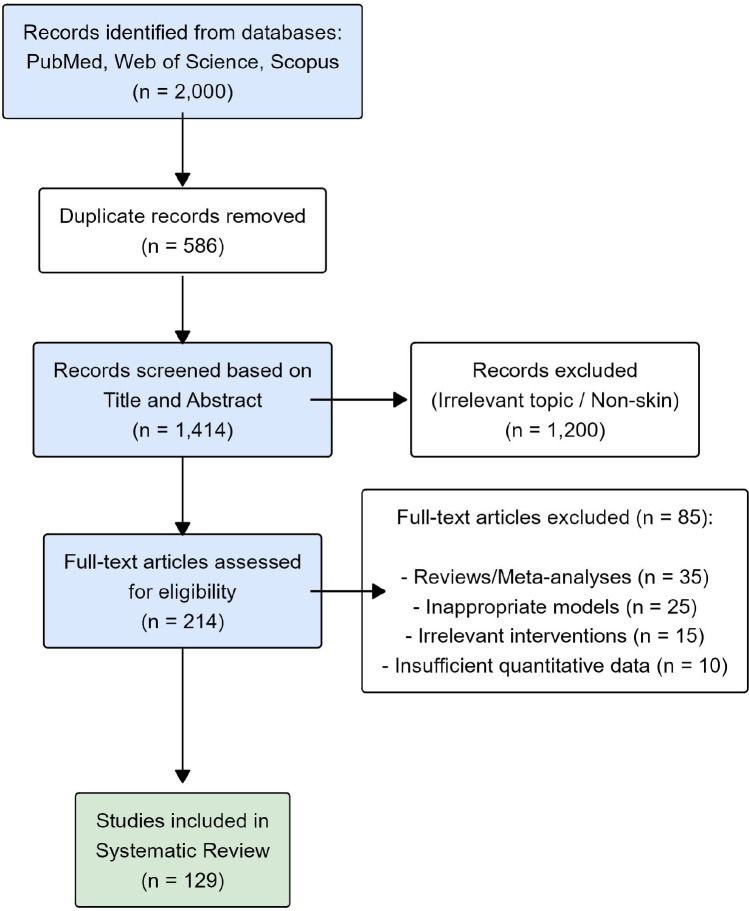
Literature identification and screening process.

A total of 2,350 records were identified through initial database searching of PubMed, Web of Science, and Scopus. The detailed search strategies are provided in **Supplementary Table 1**. After removing 650 duplicate records, 1,700 studies remained for screening based on their titles and abstracts. Of these, 1,450 records were excluded as they were irrelevant to the topic. The full texts of the remaining 250 articles were sought and successfully retrieved for detailed eligibility assessment. Upon reviewing the full texts, 103 articles were further excluded for specific reasons as detailed in **Figure 2**: 40 were reviews, meta-analyses, or editorials; 30 utilized inappropriate animal models (non-skin); 20 focused on irrelevant interventions; and 13 did not report sufficient quantitative data for analysis. Ultimately, 129 studies met all inclusion criteria. These 129 studies were assessed for methodological quality using the SYRCLE tool and were included in this systematic review.

A systematic review of 129 studies retrieved from PubMed, Web of Science, and Scopus databases reveals the prevalence of different wound etiologies modeled in pre-clinical research. The bar chart categorizes studies into three primary domains: (Left, Orange) Chronic/Diabetic Wounds represent the majority of research efforts (n=75, 51%), highlighting the critical focus on impaired healing mechanisms. (Center, Green) Acute Wounds account for 35.4% (n=52) of the included studies, typically used for testing general hemostatic or closure agents. (Right, Blue) Pathological Scarring models (including keloids and hypertrophic scars) comprise the smallest subset (n=20, 13.6%). Data represents a comprehensive synthesis of abstracts to identify the primary disease modeling focus in the field over the past decade.

The diagram proposes a hierarchical framework for selecting appropriate *in vivo* models in wound healing research. The Y-axis represents Experimental Throughput (Scalability), indicating the feasibility of large sample sizes, while the X-axis denotes Translational Relevance (Clinical Fidelity), reflecting the anatomical and physiological similarity to human skin. (Top-Left) Genetically modified mice provide high throughput and are ideal for Mechanism Discovery, utilizing tools such as the Cre-LoxP (Cyclization Recombination-Locus of X-over P1) system for gene editing. (Center) Rat models serve as an intermediate platform for Pharmacological Screening, allowing for composite models and longitudinal sampling. (Bottom-Right) Large animals (Rabbits/Pigs) exhibit low throughput but high clinical fidelity, making them essential for Clinical Validation due to their anatomical similarity and comparable scar quality to humans. The arrows illustrate the recommended translational pipeline: progressing from mechanistic exploration in mice, to screening in rats, and finally to pre-clinical validation in large animals.

### 3.2 Quality assessment and risk of bias

Assessment using the SYRCLE tool revealed a generally unclear risk of bias across the included studies. The comprehensive risk of bias summary is presented in **Supplementary Figure 1**. While 85% of studies reported random allocation of animals to treatment groups, less than 20% explicitly described the method of sequence generation (e.g., computer-generated random numbers). Notably, blinding of outcome assessors (detection bias) was reported in only 32% of studies, primarily in those utilizing histological scoring. Baseline characteristics (age, weight, sex) were well-balanced in 95% of studies. The lack of rigorous reporting on blinding and allocation concealment suggests a potential risk of overestimating therapeutic efficacy in the reviewed literature.

Review authors’ judgments about each risk of bias item presented as percentages across all 129 included studies. The assessment was performed using the Systematic Review Centre for Laboratory Animal Experimentation (SYRCLE) risk of bias tool. The stacked bar chart illustrates the proportion of studies evaluated as having a low risk of bias (green), unclear risk of bias (yellow), or high risk of bias (red) for each of the ten specific domains (e.g., sequence generation, allocation concealment, blinding). The x-axis represents the percentage of total studies, while the y-axis lists the specific bias domains assessed.

## 4 Acute skin wound models

### 4.1 Incisional wound models

Incisional skin wounds are ubiquitous in clinical practice (e.g., tumor excision, plastic and reconstructive surgery) and are typically managed by primary intention healing through wound edge apposition and suture closure (Masson‐Meyers et al., 2020). In experimental research, incisional models are widely used to (i) interrogate stage-specific mechanisms of repair, (ii) evaluate therapeutic interventions and drug-delivery strategies, and (iii) test suture materials and closure techniques. Their popularity stems from their procedural simplicity, high reproducibility, and standardized wound geometry, which together enable relatively precise comparisons across experimental groups (Li et al., 2007). However, despite this apparent simplicity, the lack of standardized reporting—specifically regarding randomization and blinding—often undermines the reproducibility of these studies. The translational relevance of incisional models is highly species-dependent, shaped by skin structure (dermal thickness, hair follicle density), immune response dynamics, biomechanical properties, and the balance between contraction and re-epithelialization. Specifically, the inherent tension of human skin, defined by Langer’s lines, is difficult to replicate in loose-skinned animals, necessitating careful orientation of incisions in animal models. These variables influence not only the macroscopic closure rate, but also the molecular programs governing inflammation resolution, fibroplasia, angiogenesis, and ultimately scar quality. Accordingly, rational model selection should be framed by the research question and the intended translational endpoint (e.g., early inflammatory signaling vs. scar formation). Below, we provide a structured overview and critical appraisal of incisional wound models in mice, rats, and rabbits, with emphasis on their advantages, limitations, and translational suitability.

#### 4.1.1 Mouse incisional wound models

Model description: The procedure is strictly standardized to minimize variability. Following anesthesia with intraperitoneal ketamine (100 mg/kg) and xylazine (10 mg/kg), the dorsal skin is shaved and depilated. Two parallel, full-thickness linear incisions (typically 2.0–2.5 cm long) are created using a sterile No. 15 scalpel blade, penetrating the epidermis, dermis, and panniculus carnosus. The wounds are immediately approximated with interrupted 6–0 nylon sutures or surgical staples to simulate primary intention healing. The distance between incisions is kept >1.5 cm to prevent vascular crosstalk (Ryan et al., 1999; Li et al., 2021). Post-operative analgesia (e.g., buprenorphine SR) is mandatory to avoid stress-induced immunosuppression (Shah et al., 2024).

Species selection: Male ICR, C57BL/KsJ, and C57BL/6 mice (male, 6–8 weeks; 18–24 g) are the premier choice due to their Th1-dominant immune response and extensive genomic mapping. The availability of transgenic strains, such as LysM-cre (myeloid lineage tracking) or Lgr6-GFP (stem cell tracking), makes this model indispensable for mechanistic studies (Dunn et al., 2013; Gao et al., 2020).

Advantages: This model offers the highest genetic tractability and cost-efficiency. It allows for high-throughput screening of specific genes involved in the initial hemostasis and inflammation phases. The linear geometry simplifies the biomechanical assessment of “breaking strength” using tensiometers.

Disadvantages: The thin dermis and the rapid contraction driven by the panniculus carnosus muscle can obscure dermal regeneration outcomes.

Clinical relevance & biochemical validation: This model is the primary platform for dissecting the early inflammatory cascade. It validates the temporal expression of pro-inflammatory cytokines (IL-1β, TNF-α, IL-6) (Lin et al., 2003) via qPCR, which typically peak at 24 hours (Lin et al., 2003; Du et al., 2018; Li et al., 2021; He et al., 2023). Crucially, it allows for the analysis of the TLR4/NF-κB signaling pathway, which initiates the immune response (Krzyszczyk et al., 2018). Researchers utilize this model to track the phenotypic switch of macrophages from pro-inflammatory M1 (iNOS+, CD86+) to pro-reparative M2 (Arg-1+, CD206+), a critical biochemical checkpoint for successful healing (Larouche et al., 2018).

Limitations: Translational gaps: immune, microbiome, & environment: The limitations of this model extend far beyond the well-known panniculus carnosus muscle.

1. Immunological divergence: Mice exhibit a neutrophil-heavy (75-90%) immune response, whereas humans have a more balanced neutrophil/lymphocyte ratio (50-70% neutrophils). This skew can lead to an exaggerated acute inflammatory phase that does not mirror human chronic inflammation.
2. Microbiome & SPF status: Most laboratory mice are Specific Pathogen Free (SPF). Their skin lacks the complex commensal microbiome (e.g., S. epidermidis, C. acnes) found in humans. This “sterile” baseline fails to mimic the “primed” immune state of human skin, which is constantly interacting with environmental microbes.
3. Housing temperature (thermal stress): Standard mouse housing (20–22 °C) is below their thermoneutral zone (30 °C). This chronic cold stress triggers norepinephrine release, which is known to systemically suppress the immune response and delay healing, a confounding factor rarely accounted for in human trials.
4. Age factor: Using 8–10 week old mice is equivalent to studying human teenagers. This ignores the senescence-associated secretory phenotype (SASP) present in the elderly population, who are the primary demographic for wound complications.

#### 4.1.2 Rat incisional wound models

Model description: Anesthesia protocols frequently include inhalational isoflurane (Gao et al., 2020) or injectable ketamine (80–100 mg/kg) combined with xylazine (≈5 mg/kg) (Petersen et al., 2016). For analgesia, buprenorphine (e.g., 0.03 mg/kg, intraperitoneal) may be administered preoperatively and continued postoperatively on a scheduled basis (Petersen et al., 2016). Some protocols additionally report non-opioid analgesia (e.g., metamizole in drinking water) and prophylactic antibiotics (e.g., enrofloxacin in drinking water) to reduce infection-related variability (Petersen et al., 2016). Longitudinal incisions ranging from ~1 to 6 cm are typically created using sterile blades (e.g., No. 15 or No. 11) or scissors, with closure using nylon or comparable sutures (e.g., 5–0 nylon; or alternative suture specifications depending on the anatomical site and study design) (Brennan et al., 1996; Petersen et al., 2016; Varadkar and Gadgoli, 2021).

Species selection: Sprague–Dawley rats (Brennan et al., 1996), Wistar rats (Petersen et al., 2016), or Wistar albino rats of either sex (Varadkar and Gadgoli, 2021) (typically 6–12 weeks; 150–600 g). Their physiological robustness makes them ideal for testing surgical devices (sutures, staples) and topical formulations that require absorption kinetics similar to humans.

Advantages: Rats offer a larger operating field, which facilitates repeated sampling (tissue, exudate), serial imaging, and multi-dose pharmacologic regimens. Their skin structure and immune response are often regarded as closer to humans than mice, contributing to improved stability for some readouts (Chen et al., 2018). Specifically, the larger skin surface area allows for the extraction of sufficient tissue for simultaneous biomechanical testing (breaking strength strips) and histological analysis (hydroxyproline content for collagen quantification) from the same animal, reducing the total number of animals required. These features make rats a practical intermediate platform for studies that require more extensive perioperative management or richer longitudinal observation. The rat model bridges the gap between molecular murine studies and large animal translational trials. Its key advantage is the tissue volume, which permits simultaneous biomechanical, histological, and biochemical analysis from a single animal (reducing the 3Rs burden).

Disadvantages: Higher husbandry costs and lower throughput than mice. The choice of suture material is a critical variable often overlooked; multifilament sutures (like silk) can induce a more robust foreign body reaction compared to monofilament sutures (like nylon), potentially skewing inflammatory readouts and collagen deposition rates. Although the dermis is thicker than in mice, healing still relies partially on contraction unless splinted or sutured under tension. The endpoints such as “time to closure” can still be disproportionately influenced by contractile responses rather than epithelial regeneration. This is not a minor methodological detail: if a therapy primarily modulates fibroblast contractility, it may appear highly effective in rodents yet translate poorly to humans where the relative contribution of contraction is lower.

Clinical relevance & biochemical validation: Rat incisional models are particularly useful for mechanistic studies spanning the proliferation and early remodeling phases, including fibroblast proliferation, collagen deposition, MMP-9 expression, and angiogenesis-associated programs (e.g., VEGF/VEGFR axis; HIF-1α–linked pathways) (Dai et al., 2011; Latrach et al., 2022). Additionally, when deeper incisions or specific anatomical sites are used to better reflect clinical wound complexity, the model may better approximate certain clinical scenarios (Brennan et al., 1996). This model focuses on the Proliferative and Remodeling phases. It is the gold standard for quantifying Collagen Maturation. Biochemically, it validates the ratio of Type I (mature) to Type III (immature) collagen using Picrosirius Red staining under polarized light. A higher Type I/III ratio correlates with recovered tensile strength. Furthermore, it allows for the zymographic analysis of Matrix Metalloproteinases (MMP-2 and MMP-9) activity, which regulate ECM turnover. The measurement of Hydroxyproline content (a direct surrogate for total collagen) in this model provides the quantitative data necessary to claim “improved structural integrity” (Raziyeva et al., 2021).

Limitations: Translational gaps: immune, microbiome, & environment:

1. Fibroblast heterogeneity: Rat fibroblasts proliferate much more rapidly than human fibroblasts. This intrinsic cellular difference means rats can overcome wound defects that would be chronic in humans, potentially yielding “false positive” results for wound healing accelerants.
2. Microbial ecology: The rat skin microbiome differs significantly from humans, particularly in the abundance of Staphylococcus species. Since the microbiome educates the resident T-cells (Trm), the local immune response to an incision in a rat is fundamentally different from a human surgical site.
3. Metabolic rate: The high metabolic rate of rats accelerates the clearance of systemic drugs. A topical treatment that lasts 12 hours in humans might be metabolized or physically rubbed off by a grooming rat in 2 hours, complicating dosing regimens.
4. Age bias: Most studies use young adult rats (12 weeks). However, the decline in collagen density and skin thickness associated with aging (atrophy) is a critical factor in wound dehiscence that is missed in these young models.

#### 4.1.3 Rabbit incisional wound models

Model description: Anesthesia is typically achieved via intramuscular regimens that may include acepromazine (≈1 mg/kg) with ketamine (20–50 mg/kg) (Latrach et al., 2022), or ketamine-based combinations incorporating xylazine (e.g., ~5 mg/kg) (Sarojini et al., 2021). After aseptic preparation and shaving of the lateral thigh region, full-thickness cutaneous incisions of approximately 3–10 cm are created using sterile blades (e.g., No. 24) (Sarojini et al., 2021). Wounds are closed with absorbable braided sutures on triangular curved needles or with 3-0/4–0 sutures using simple interrupted patterns (Dai et al., 2011; Latrach et al., 2022). Postoperative analgesia commonly includes buprenorphine (e.g., 0.01 mg/kg, IM) and/or fentanyl patches, with free access to food and water to minimize stress-related confounding (Sarojini et al., 2021).

Species selection: Adult New Zealand White rabbits (often ~1 year old; ~2.5–3.6 kg) are commonly used (Dai et al., 2011; Latrach et al., 2022).

Advantages: Rabbit skin—particularly with respect to dermal thickness and vascular distribution—can more closely approximate human cutaneous biology than rodent skin. Critically, rabbit wound repair relies more strongly on re-epithelialization rather than pure contraction, improving fidelity for studies focused on epithelial dynamics, dermal remodeling, and scar maturation (Latrach et al., 2022). Moreover, rabbit incisional models are reported to form hypertrophic scars with morphological similarity to human hypertrophic scar tissue, which is a major advantage when scarring is the primary translational endpoint (Tunca et al., 2019; Mistry et al., 2022). Specifically, the rabbit ear hypertrophic scar model is distinct from dorsal models; the tight adherence of skin to the underlying cartilage in the ear minimizes contraction, forcing healing to occur via granulation tissue formation and re-epithelialization, thereby creating a highly relevant platform for testing anti-scarring therapeutics using metrics like the Scar Elevation Index (SEI).

Disadvantages: Rabbits are stress-sensitive; the model requires advanced surgical skills and higher costs (Mistry et al., 2022).Inter-individual variability in scarring response can be high, necessitating larger sample sizes (n>6 per group).

Clinical relevance & biochemical validation: Therefore, rabbit incisional models are best positioned as a validation and mechanistic platform for scar formation and late remodeling, including targets linked to fibrotic pathways and scar biology (e.g., TSG-6–related processes) (Nabai and Ghahary, 2017a), rather than as a first-line discovery model. This model validates the Fibrotic Signaling Pathways discussed in the introduction. Specifically, it demonstrates the sustained upregulation of Transforming Growth Factor-β1 (TGF-β1) and the phosphorylation of Smad2/3, which drive the differentiation of fibroblasts into α-SMA positive myofibroblasts. Biochemical analysis often includes Western Blotting for Connective Tissue Growth Factor (CTGF) and Fibronectin, markers that are persistently elevated in human hypertrophic scars. This biochemical profile makes the rabbit model the standard for testing anti-scarring therapies (e.g., corticosteroids, siRNA) (Mistry et al., 2022).

Limitations: Translational gaps: immune, microbiome, & environment:

1. Immunological sensitivity: Rabbits are highly sensitive to stress-induced catecholamines, which can cause profound vasoconstriction in the ear (an end-organ), artificially inducing ischemia and altering healing kinetics unpredictably compared to humans.
2. Anatomical discrepancy: The rabbit ear lacks subcutaneous fat (hypodermis). In humans, the hypodermis plays a crucial role in providing stem cells (adipose-derived stem cells) and cytokines (adipokines) during healing. The absence of this layer in the ear model removes a key biological compartment present in human surgical wounds.
3. Housing conditions: Rabbits housed singly (to prevent fighting/ear damage) suffer from social isolation stress, which has been shown to elevate cortisol levels and delay barrier recovery, introducing a “psychological” confounder.

### 4.2 Excisional wound models

Excisional wounds represent a distinct clinical entity from simple surgical incisions. They typically involve substantial tissue loss and may arise in settings such as extensive burns, chronic ulcerations, traumatic tissue loss, and major resections, where delayed closure and impaired regeneration are common. Experimentally, excisional wounds are created by removing a full-thickness segment of skin (epidermis, dermis, and often subcutaneous fat), generating a standardized tissue deficit that allows controlled investigation of re-epithelialization, granulation tissue formation, angiogenesis, extracellular matrix remodeling, and scar outcomes. Unlike incisional wounds where tensile strength is the primary outcome, excisional models require rigorous quantification of wound closure rates using digital planimetry and ImageJ software to calculate the percentage of wound area reduction over time. From a translational standpoint, the major methodological challenge in rodents is that wound closure can be dominated by contraction (owing to the panniculus carnosus), which may overestimate interventions that primarily enhance contractile closure rather than epithelial regeneration—an important consideration when extrapolating to human wound repair.

#### 4.2.1 Mouse excisional wound models

Model description: Under aseptic conditions, anesthesia is induced via intraperitoneal pentobarbital sodium (Rhea and Dunnwald, 2020), chloral hydrate (10%; 0.003 mL/g) (Yang et al., 2020), or ketamine (≈80 mg/kg) combined with diazepam (≈5 mg/kg) (Lambebo et al., 2021). Full-thickness excisional wounds are created on the dorsal skin, frequently as bilateral paired wounds to facilitate within-animal comparison. Reported wound sizes include 6–8 mm circular defects (Shah et al., 2024) or larger standardized areas (e.g., ~300 mm²) depending on study objectives, generated using sterile biopsy punches/perforators (Rhea and Dunnwald, 2020; Yang et al., 2020) or sharp sterile scissors (Ahmad et al., 2021).

Species selection: ICR, BALB/c, Swiss albino mice, and type 2 diabetes mellitus (T2DM) models (typically 6–10 weeks; 18–30 g) (Lima et al., 2017; Rhea and Dunnwald, 2020; Yang et al., 2020; Lambebo et al., 2021).

Advantages: The mouse excisional model is a tool for mechanistic research because it supports large cohort sizes, genetic manipulation, and systematic pathway interrogation at relatively low cost (Zomer and Trentin, 2018). It is particularly useful when the objective is to dissect early inflammatory programs, cytokine networks, macrophage phenotypes, and angiogenic signaling, or when screening candidate compounds prior to validation in larger species (Rodrigues et al., 2019). Additionally, the availability of transgenic mice expressing fluorescent markers (e.g., LysM-GFP for neutrophils) allows for real-time *in vivo* imaging of immune cell dynamics during the healing process. Combines mouse genetic tools with human-like healing mechanics. It is ideal for digital planimetry to calculate the % Wound Closure rate and for assessing the “Epithelial Gap” in histology (Mujahid et al., 2025).

Disadvantages: The trap of contraction-based closure. A fundamental translational flaw is that conventional dorsal excisional wounds in mice close primarily through rapid contraction, often creating a “purse-string” effect that masks the true efficacy of agents designed to promote re-epithelialization (Abdullahi et al., 2014). Recent comparative studies emphasize that while murine wounds may visually close within 14 days, the underlying collagen organization often remains immature compared to human scars (Zomer and Trentin, 2018). We strongly caution readers against interpreting wound area reduction as a standalone metric of success. Unless the specific goal is to study contracture, data derived from non-splinted mouse wounds should be substantiated by quantitative histology—specifically measuring the neo-epithelial tongue length rather than the open wound area—to avoid misleading conclusions regarding epithelial regeneration (Crane et al., 2020). Small wound size limits the volume of wound fluid (exudate) available for proteomic analysis.

Clinical relevance & biochemical validation: This model is the benchmark for studying Angiogenesis and Re-epithelialization. Biochemically, it allows for the quantification of Vascular Endothelial Growth Factor (VEGF) and HIF-1α levels in the granulation tissue, linking hypoxia to vascular repair (Ruthenborg et al., 2014). Immunohistochemistry for CD31 (PECAM-1) allows for the counting of capillary loops. Furthermore, the model validates the expression of Keratin 6, 16, and 17, which are markers of the “activated” keratinocyte phenotype required for migration, distinguishing them from the basal Keratin 14 (Prathiba et al., 2001).

Limitations: Translational gaps: immune, microbiome, & environment:

1. Hair cycle influence: Mouse skin contains a dense array of hair follicles. Since hair follicles are rich reservoirs of stem cells (Lgr5+, Lgr6+), wounds in mice—even splinted ones—recruit stem cells much more efficiently than human skin, which is relatively hair-sparse (interfollicular epidermis). This leads to an overestimation of regenerative capacity.
2. Immune naivety: Laboratory mice live in ultra-hygienic bubbles (IVC cages). Their immune system is “naive” (Th0). In contrast, adult human skin has a “memory” immune system (Tissue Resident Memory T cells) shaped by decades of pathogen exposure. Therapies that work by boosting immunity in naive mice often fail in humans who already have chronic, dysregulated inflammation.
3. Age: chronic wounds (e.g., venous leg ulcers) are diseases of aging. Using 8-week-old mice to model these conditions fails to capture the senescence of fibroblasts and the exhaustion of stem cells that characterize the actual clinical problem.

The splinted (ring-restricted) excisional model: why it is favored, and what it still cannot solve. To mitigate contraction bias, modified approaches employ mechanical splinting, most commonly by securing a rubber or silicone ring around the wound perimeter to restrict skin movement. By limiting contraction, the model shifts the dominant closure mechanism toward re-epithelialization, cell proliferation, granulation tissue growth, and angiogenesis, thereby improving alignment with key biological features of human wound healing (Dunn et al., 2013). In practice, this modification often strengthens the interpretability of endpoints such as epithelial gap closure, neo-vascular density, and matrix deposition patterns. However, splinting is not a universal remedy and should be framed critically: Rings and fixation methods can introduce local mechanical stress, foreign-body reactions, or micro-injury at suture points, which may confound inflammatory readouts and angiogenesis measures. Researchers must also be vigilant about device failure, as mice frequently chew or dislodge the splints, leading to high dropout rates or the need for single-housing, which in turn induces social stress. These effects can be particularly problematic when the intervention being tested also targets immune modulation or vascular responses. Even with contraction restriction, differences in epidermal thickness, hair follicle density, and immune kinetics persist between mice and humans, and these may influence keratinocyte migration, macrophage dynamics, and cytokine trajectories (Martin, 1997). Healing rate, immune polarization tendencies, and contraction propensity differ across strains (e.g., C57BL/6 versus BALB/c), affecting reproducibility and complicating cross-study comparisons if strain choice is not justified and controlled (Diegelmann and Evans, 2004). For this reason, strain selection should be treated as an experimental variable rather than a trivial logistical choice.

#### 4.2.2 Excisional wound models in rats

Model description: General anesthesia is most commonly achieved with inhalational isoflurane (≈4%) (Mendes et al., 2022), while earlier protocols have also reported chloroform anesthesia (100 ppm; v/v) (Kuma et al., 2022). After dorsal shaving under anesthesia, full-thickness circular wounds (approximately 0.5–4 cm²) are created using sterile biopsy punches (Varadkar and Gadgoli, 2021; Mendes et al., 2022) or by excising tissue along predefined outlines with toothed forceps, scalpel blades, and pointed scissors (Kuma et al., 2022). To improve inter-animal consistency, the wound margins are often traced with a circular stainless-steel template and methylene blue prior to excision (Kuma et al., 2022).

Species selection: Wistar albino or Sprague–Dawley male rats (8–10 weeks, 150–200 g) (Varadkar and Gadgoli, 2021; Mendes et al., 2022).

Advantages: Compared with mice, rats offer an expanded operative field that facilitates more reliable wound creation, repeated sampling, and longer-term follow-up, which is particularly relevant when the intended readouts extend beyond early closure (e.g., matrix remodeling, scar maturation, and biomechanical properties). This practical advantage makes rat excisional models more suitable for mechanistic studies spanning the proliferative-to-remodeling transition, where tissue heterogeneity and sampling depth become limiting factors in smaller rodents. Furthermore, the volume of granulation tissue generated in rats is sufficient for detailed biochemical analysis, such as hydroxyproline assays to quantify total collagen content, which provides a robust quantitative measure of fibrosis.

Disadvantages: Significant contraction occurs if not splinted; requires large amounts of test drug.

Clinical relevance & biochemical validation: A principal translational caveat is that dorsal rat wounds, similar to mouse wounds, are strongly influenced by panniculus carnosus–associated contraction, which can artificially accelerate macroscopic closure. This feature has two implications that should be explicitly acknowledged in a review: “Faster closure” in dorsal rat wounds may primarily reflect enhanced contractility rather than improved epithelial repair or dermal regeneration; and interventions that mainly modulate fibroblast contractility or myofibroblast activity may appear disproportionately effective in rodents relative to humans. Accordingly, when the scientific aim is to approximate human-like healing dynamics, mechanical restraint (e.g., rubber/silicone ring splinting) is often introduced to suppress contraction and increase the relative contribution of re-epithelialization and granulation tissue formation. Notably, splinting improves interpretability for epithelial and angiogenic endpoints, but it can also introduce device-related inflammation and mechanical artifacts that confound immune readouts—an issue that should be considered when evaluating anti-inflammatory therapies. This larger wound bed in rats also makes them an ideal platform for studying infected wounds, allowing for the stable inoculation of bacterial biofilms (e.g., *Staphylococcus aureus* or Pseudomonas aeruginosa) to test antimicrobial dressings. Collectively, these steps yield a model with relatively controllable geometry and adequate tissue volume for serial histology and molecular assays. Despite contraction bias, rat excisional wounds can generate scar phenotypes with clinically informative features. At approximately 12 weeks post-wounding, rat scar tissue has been reported to display irregular, spiral-patterned collagen organization reminiscent of human hypertrophic scars (Mistry et al., 2022). Histological assessment using Masson’s Trichrome or Picrosirius Red staining under polarized light is essential in these studies to visualize collagen fiber orientation and maturity. Moreover, under high mechanical strain, rat scar tissue exhibits expression patterns of TGF-β1 and α-SMA that align closely with those observed in human hypertrophic scars (Gushiken et al., 2022). These observations support the use of rat systems for studying mechanically regulated fibrosis, particularly when the endpoint is not merely closure time but matrix architecture and myofibroblast-driven remodeling. This model is essential for analyzing the Oxidative Stress and Protease Environment. It validates the dysregulation of the MMP-9/TIMP-1 balance, a hallmark of chronic wounds. Biochemical assays for Myeloperoxidase (MPO) activity provide a quantitative measure of neutrophil infiltration and inflammation (Gibson and Schultz, 2013). Additionally, markers of oxidative damage, such as Malondialdehyde (MDA) and Superoxide Dismutase (SOD) activity, are routinely measured to evaluate the antioxidant capacity of novel wound dressings (Wilgus et al., 2013).

Limitations: Immune, microbiome, & environment:

1. Microbiome specificity: Rat skin flora does not naturally contain *S. aureus* or P. aeruginosa, the two main pathogens in human chronic wounds. Inoculating these bacteria creates an acute invasion model, not a chronic colonization model. The host-pathogen interaction is “forced” rather than established over time.
2. Environment (bedding): Rats are in constant contact with bedding material (sawdust/paper), which introduces foreign body contaminants into open wounds that are not present in human ulcers protected by footwear or clothing. This can induce a foreign body giant cell reaction that confounds histological scoring.
3. Growth phase: Rats have indeterminate growth (they grow throughout their life). This intense systemic anabolic drive promotes tissue deposition in wounds much more aggressively than in humans, who have ceased somatic growth.

#### 4.2.3 Excisional wound models in rabbits

Model description: General anesthesia is typically induced by intramuscular ketamine (45 mg/kg) combined with toluene thiazide (5 mg/kg) (Guoqi et al., 2018; Dolivo et al., 2023). The auricular region is shaved and disinfected (often twice with 70% ethanol), followed by local anesthesia according to protocol (e.g., infiltration with 1% lidocaine; alternative regimens have also been reported) (Guoqi et al., 2018; Gushiken et al., 2022; Dolivo et al., 2023). Using scalpels or sterile blades, investigators usually create six standardized full-thickness dermal excisions (6–8 mm in diameter) on the ventral surface of the ear (Guoqi et al., 2018; Dolivo et al., 2023). Crucially, the epidermis, dermis, and perichondrium must be removed to expose the underlying cartilage, as this rigid base is what physically prevents wound contraction. The planar anatomy and accessibility of the ear facilitate consistent wound placement and longitudinal assessment.

Species selection: Male or female Japanese white rabbits (12–24 weeks old; 1.5–5.0 kg) (Guoqi et al., 2018; Dolivo et al., 2023).

Advantages: A defining strength of the rabbit ear excisional model is its closure biology, which is more comparable to human cutaneous repair than conventional dorsal rodent excisional wounds. In contrast to mouse and rat dorsal wounds—where panniculus carnosus–associated contraction can dominate—rabbit ear wounds exhibit minimal contraction and heal primarily via granulation tissue formation and re-epithelialization, processes central to human wound healing (Nabai and Ghahary, 2017b). This feature improves interpretability for endpoints related to epithelial migration, granulation quality, angiogenesis, and matrix remodeling, and reduces the risk of overestimating therapies that mainly accelerate contraction. The rabbit ear model is also widely used in pathological scarring research. Scar histology has been described to show features consistent with hypertrophic scarring, including increased fibroblast cellularity and disorganized collagen architecture, supporting its frequent use for mechanistic studies of scar formation and preclinical evaluation of anti-scar interventions (Nabai and Ghahary, 2017b). To quantitatively evaluate scarring severity in this model, the Scar Elevation Index (SEI) is the gold standard metric. The SEI represents the ratio of the total scar tissue height to the height of the surrounding normal tissue; an SEI > 1.0 indicates hypertrophic scar formation, providing a precise, objective endpoint for comparing therapeutic efficacy. Importantly, the bilateral ear configuration enables within-animal controlled designs, which can substantially reduce inter-individual variability and strengthen statistical power in intervention studies (Dolivo et al., 2023). From a practical standpoint, rabbits are readily available and tractable for controlled experimental work, with established husbandry and traceability resources (Mistry et al., 2022).

Disadvantages: Despite its translational strengths, the rabbit ear model has constraints that should be explicitly acknowledged in a review. First, the auricular microenvironment (e.g., cartilage adjacency, regional biomechanics, and vascular distribution) differs from many clinically relevant sites, which may influence fibroblast activation and collagen organization in a site-specific manner. Second, the typical wound size (6–8 mm) is experimentally convenient but may not capture scaling effects pertinent to large tissue-loss wounds or chronic ulcer beds. Third, heterogeneity in local anesthetic regimens and perioperative handling across studies can introduce non-trivial variability, complicating cross-study comparisons unless protocols are carefully harmonized (Guoqi et al., 2018; Gushiken et al., 2022; Dolivo et al., 2023). Accordingly, model selection and outcome interpretation should be endpoint-driven, with emphasis on epithelialization and scar remodeling metrics rather than contraction-dependent closure rates. The thin skin over cartilage lacks the subcutaneous fat and cushioning found in most human pressure ulcers.

Clinical relevance & biochemical validation: Given its contraction-minimized healing pattern and scar-informative histology, the rabbit ear excisional model is particularly suitable for (i) mechanistic investigations of hypertrophic scar formation, (ii) efficacy evaluation of emerging anti-scar interventions (Dolivo et al., 2023), and (iii) profiling of fibrosis- and inflammation-associated mediators (e.g., TNF, TGF-β1, MMPs, IL-6) across the granulation and remodeling phases (Nabai and Ghahary, 2017a). Overall, compared with contraction-prone dorsal rodent excisional wounds, the rabbit ear model provides a contraction-minimized platform in which epithelialization-, granulation-, and scar remodeling–related endpoints can be interpreted with greater translational fidelity, albeit with anatomical site–specific constraints that should be considered when extrapolating to human clinical wounds (Nabai and Ghahary, 2017b). This model is used to validate Extracellular Matrix (ECM) deposition (Tracy et al., 2016). Researchers quantify the expression of Fibronectin, Vitronectin, and Collagen III in the newly formed tissue. It is also a key model for studying Connexin 43 (Cx43) gap junction dynamics; Cx43 downregulation at the wound edge is a biochemical marker of successful migration, which can be modulated by therapeutic peptides (Grek et al., 2015).

Limitations: Translational gaps: immune, microbiome, & environment:

1. Regenerative vs repair: Rabbits have a higher propensity for regeneration (e.g., ear holes can sometimes close with cartilage regeneration) compared to humans who heal strictly by scarring. This biological “optimism” of the rabbit model can overstate the regenerative potential of biomaterials.
2. Immunological “hygiene hypothesis”: Like rodents, lab rabbits are kept clean. They lack the chronic sub-clinical fungal or bacterial burdens (e.g., Tinea pedis) that many elderly patients have, which perpetually stimulate skin immunity (TLR activation).
3. Circadian rhythm: Rabbits are crepuscular/nocturnal. Most experiments and dosing occur during the day (their sleep phase), potentially disrupting circadian clock genes (Per/Cry) in the wound, which are now known to regulate actin dynamics and cell migration.

### 4.3 Thermal wound models

Thermal injury represents the predominant etiology of burn trauma in humans; a prior synthesis reported that thermal mechanisms account for the majority of burn cases (commonly cited around ~86%), far exceeding electrical and chemical causes (Smolle et al., 2017). From a modeling perspective, thermal burns are attractive because they allow controlled manipulation of temperature–time exposure, generating graded tissue necrosis and enabling mechanistic interrogation of inflammatory amplification, microvascular dysfunction, and subsequent repair. Unlike excisional wounds where tissue is immediately removed, thermal injuries retain necrotic tissue (eschar), which serves as a reservoir for inflammatory mediators and a potential nidus for infection, thereby mimicking the complex pathophysiology of clinical burns more accurately.

#### 4.3.1 Thermal wound models in mice

Model description.: Animals are anesthetized using intraperitoneal ketamine-based regimens (e.g., ketamine alone or ketamine combined with lidocaine for sedation) (Karimi et al., 2014; Lambebo et al., 2021) or inhalational isoflurane (e.g., ~2.5%) (Vinaik et al., 2020). Following dorsal hair removal and antisepsis (commonly 70% ethanol) (Lambebo et al., 2021), peri-procedural supportive care is often included—such as subcutaneous lactated Ringer’s solution to mitigate dehydration and reduce procedure-related stress, and buprenorphine for analgesia (Vinaik et al., 2020). Adequate analgesia is not only an ethical imperative but also a scientific necessity, as uncontrolled pain triggers a systemic stress response that can significantly alter immune cell function and wound healing kinetics. Several standardized approaches: (1)Molten beeswax contact burn: heated beeswax (e.g., ~80 °C) is poured into a metal cylinder that is briefly applied to the dorsum (e.g., ~8 s), leaving a circular full-thickness–predominant lesion (Lambebo et al., 2021). (2)Heated metal probe contact burn: a defined probe (e.g., 1.5 × 1.5 cm) heated in near-boiling water (e.g., ~96 °C) is applied to the dorsum for a fixed duration (e.g., ~8 s) to generate a standardized deep burn (often described as third-degree) (Karimi et al., 2014). (3)Hot-water immersion scald model: immersion of the dorsum in near-boiling water (e.g., ~98 °C for ~10 s) can generate a larger TBSA injury (e.g., 15–20% TBSA), sometimes combined with brief abdominal exposure to create a second site of injury depending on experimental aims (Vinaik et al., 2020).

Species selection: Swiss albino, male inbred BALB/c, and wild-type C57BL/6 mice (typically 6–10 weeks old, 20–40 g) (Karimi et al., 2014; Vinaik et al., 2020; Ahmad et al., 2021).

Advantages: Murine thermal burn models are particularly valuable for mechanistic discovery, because strain diversity and genetic tools allow causal testing of immune and repair pathways (e.g., neutrophil/macrophage dynamics, cytokine programs, oxidative stress responses) across tightly controlled injury parameters (Vinaik et al., 2020; Lambebo et al., 2021). Their low cost and scalability make them suitable for early-stage screening of candidate therapeutics before validation in higher-fidelity systems (Vinaik et al., 2020; Lambebo et al., 2021). Importantly, contact/probe approaches provide relatively high reproducibility for localized burn biology, supporting standardized comparisons of inflammatory cell infiltration, angiogenesis, and re-epithelialization under defined conditions (Martin, 1997). Furthermore, the presence of the eschar in these models allows researchers to study the efficacy of enzymatic debridement agents and topical antimicrobials in penetrating necrotic tissue.

Disadvantages: Mouse skin differs from human skin in epidermal thickness, adnexal density, and subcutaneous structure, which can alter keratinocyte behavior, appendage-derived re-epithelialization, and scarring trajectories (Martin, 1997). Thus, findings on regeneration or scar quality may not extrapolate linearly to human burns. As in other murine cutaneous wounds, contraction can contribute disproportionately to apparent “closure,” potentially inflating efficacy for interventions that primarily modulate contractility rather than epithelial restoration. Even within “standardized” protocols, small variations in contact pressure, device temperature stability, and exposure time can shift burn depth. This matters because partial-thickness versus full-thickness injuries recruit distinct repair programs and differ in risk of burn wound conversion. To address this variability, histological verification using lactate dehydrogenase (LDH) staining to mark viable cells or Laser Speckle Contrast Imaging (LSCI) to assess perfusion is recommended to objectively define burn depth. Small-area dorsal burns are well suited for local inflammatory and repair readouts but have limited capacity to model systemic burn physiology (hypermetabolism, systemic inflammation, multi-organ effects). TBSA scald models address systemic questions more directly, but at the cost of increased variability and greater demands on resuscitation/analgesia (Vinaik et al., 2020).

Clinical relevance & biochemical validation: Accordingly, mouse thermal burn models are best positioned for (i) early inflammatory mechanisms after thermal injury, (ii) gene- and pathway-level regulation of burn repair, and (iii) preliminary drug screening under controlled injury conditions (Martin, 1997; Vinaik et al., 2020). Claims about clinical translation should be framed cautiously and, where the endpoint is systemic physiology or long-term scar quality, supported by validation in models with more human-like skin structure and biomechanics. To validate mechanistic pathways, researchers should track the DAMPs-mediated Inflammatory Response. Key markers include the release of High Mobility Group Box 1 (HMGB1) and the activation of the NLRP3 Inflammasome (evidenced by Caspase-1 cleavage and IL-1β secretion). Furthermore, the upregulation of Heat Shock Protein 70 (Hsp70) serves as a critical molecular biomarker for cellular stress tolerance and survival in the penumbra zone (Vinaik et al., 2020).

Limitation: Immune, microbiome, & environment:

Hypermetabolic vs. hypothermic: Following severe burns, humans enter a “hypermetabolic flow phase” (catabolism). Mice, due to their high surface-area-to-volume ratio, are prone to rapid hypothermia. Unless housed at thermoneutrality (30 °C), the mouse response is driven by cold stress, not burn hypermetabolism.

Panniculus contraction: The rapid contraction of the panniculus carnosus muscle (up to 80% of closure) masks the efficacy of re-epithelialization therapies.

Genomic storm: The genomic response to trauma in mice differs significantly from humans. For instance, the timing and magnitude of the “cytokine storm” (IL-6, TNF-α) in mice often do not correlate with human outcomes.

#### 4.3.2 Thermal wound models in rats

Model description: Anesthesia is commonly induced and maintained with ketamine-based regimens (e.g., ketamine 50 mg/kg combined with toluthiazide 5 mg/kg) (Cai et al., 2014) or with 3% pentobarbital sodium (e.g., 10 mg/kg) (Zhang et al., 2014; Li et al., 2017; Zhang et al., 2021). Dorsal hair removal is performed either mechanically (scissors) (Cai et al., 2014) or chemically (e.g., 20% depilatory cream) (Zhang et al., 2021), followed by antisepsis with povidone-iodine and 70% isopropyl alcohol to reduce procedure-related contamination (Cai et al., 2014). These steps are broadly consistent with efforts to standardize perioperative stress and infection risk—two factors that can otherwise confound burn-associated inflammatory readouts. Two main induction paradigms and what they model. (1) Local standardized contact burns (device-mediated heat transfer). A widely used approach employs cylindrical metal rods or solid aluminum bars (≈1.0 cm diameter) pre-heated in boiling water (≈100 °C) and applied to the dorsal skin for 10–15 seconds to generate reproducible focal thermal injuries (Cai et al., 2014; Krstic et al., 2023). This paradigm is most appropriate when the experimental objective is to interrogate local burn wound biology (e.g., inflammatory infiltration, microvascular injury, re-epithelialization, and granulation) under a relatively constrained and repeatable geometry. Although temperature and time are nominally controlled, the delivered thermal dose remains sensitive to contact pressure, rod thermal mass, dwell-time precision, and local skin thickness/perfusion, which can shift injury depth and thereby alter downstream repair programs. In a review context, this matters because “third-degree” classification in rodents is not solely a function of set temperature; small procedural differences can change the balance between coagulative necrosis, burn conversion, and viable dermal appendage retention, all of which influence epithelialization and scarring trajectories. (2) Large-area flame burns (template-defined exposure under combustion). To model clinically relevant flame injuries and larger TBSA involvement, rats may be positioned in a prefabricated template with rectangular openings that expose a defined dorsal area (commonly standardized to ~2 cm × 4 cm) (Nikahval et al., 2020). A fixed volume of mixed fuel (e.g., 0.8 mL containing gasoline, 95% alcohol, rosin, glycerin, and xylene) is applied, ignited for a set duration (e.g., 20 seconds), and extinguished rapidly (e.g., with damp cloth) (Zhang et al., 2021). This paradigm is conceptually attractive for studying burn size–dependent systemic responses, including inflammatory amplification, fluid shifts, and organ-level consequences, because it increases injury burden relative to focal contact burns (Nikahval et al., 2020; Zhang et al., 2021). The mixed-fuel formulation has an inherently complex composition and may introduce chemical irritation/toxicity in addition to thermal injury, thereby confounding attribution of biological effects to heat alone. This is not merely a theoretical concern: aromatic solvents and combustion by-products can modify local inflammation, vascular permeability, and tissue toxicity, potentially altering cytokine profiles and repair kinetics independent of thermal dose. Therefore, when such flame models are reviewed or compared across studies, it is essential to treat them as thermo-chemical injury systems unless strong controls demonstrate that chemical effects are negligible.

Species selection: Wistar albino rats (approximately 2 months old; 250–325 g ± 25 g) (Cai et al., 2014; Krstic et al., 2023).

Advantages: Rat burn models offer a practical intermediate between murine discovery systems and large-animal platforms. Their body size facilitates more precise burn-area standardization, serial sampling, and physiological monitoring, and the availability of both focal (contact) and larger-area (template/flame) paradigms allows investigators to align model choice with the intended biological question—from localized wound repair to systemic inflammatory responses (Zhang et al., 2014; Li et al., 2017; Zhang et al., 2021). Additionally, the larger wound area in rats allows for the inoculation of specific bacterial strains (e.g., Pseudomonas aeruginosa) to create burn-wound infection models, which are critical for testing novel antimicrobial dressings and understanding the interplay between thermal injury and biofilm formation.

Disadvantages: Across paradigms, two limitations should be emphasized for translational interpretation: Dose delivery variability (especially in contact burns), where pressure/thermal mass/dwell-time can shift depth and reduce cross-study comparability; and Model purity (especially in flame burns using mixed fuels), where chemical components may act as hidden variables that distort inflammatory and reparative endpoints (Zhang et al., 2021). In addition, compared with mice, rats entail higher housing costs and lower throughput, constraining their utility for large-scale screening, particularly when multi-arm intervention studies are planned.

Clinical relevance & biochemical validation: Taken together, focal rod-mediated burns are best suited for mechanistic interrogation of local burn wound repair under relatively standardized geometry, while template-defined larger-area burns are more suitable for studying how burn area and injury burden shape systemic inflammatory responses and downstream outcomes (Nikahval et al., 2020; Zhang et al., 2021). For manuscripts emphasizing clinical translation, it is advisable to align claims with the model’s dominant injury drivers (pure thermal vs thermo-chemical), and to prioritize outcome measures that match the chosen paradigm (e.g., epithelialization and granulation endpoints for contact burns; systemic cytokine kinetics and organ-level biomarkers for larger-area injuries). This model is essential for validating the Oxidative Stress Pathway. Efficacy is defined by the reduction of Lipid Peroxidation products (MDA) and the restoration of Glutathione (GSH) reservoirs in tissue and plasma (Cano Sanchez et al., 2018). Additionally, measuring Myeloperoxidase (MPO) activity provides a quantitative assessment of neutrophil infiltration, distinguishing between a healthy inflammatory response and pathological tissue damage (Peiseler and Kubes, 2019).

Limitation: Immune, microbiome, & environment:

1. Fluid resuscitation scaling: The fluid volume required to resuscitate rats (ml/kg) does not scale linearly to the human “Parkland Formula,” complicating the modeling of fluid creep and compartment syndromes.
2. Hair cycle depth: The depth of hair follicles in rats varies significantly with the hair growth cycle (Anagen vs. Telogen), which can alter the depth of the burn injury and the availability of stem cells for re-epithelialization more drastically than in humans.

#### 4.3.3 Thermal wound models in rabbits

Model description: Anesthesia is induced either by inhalational isoflurane (Shi et al., 2020) or by intramuscular administration of ketamine hydrochloride (≈35 mg/kg) combined with toluene thiazide (≈4 mg/kg) (Henke et al., 2005). Hair removal is performed with depilatory cream on the dorsum (Shi et al., 2020) or by shaving the ventral region with scissors, depending on the wound site selected (Henke et al., 2005). Thermal wounds are generated using contact-based heat transfer devices, with three frequently reported paradigms: (i) application of 3–5 layers of gauze preheated in boiling water (Shi et al., 2020), (ii) circular metal plates (≈10–22 mm diameter) heated over an open flame (nominally ~100 °C) (Henke et al., 2005; Mekkaoui et al., 2021), or (iii) preheated aluminum stamps (e.g., ~4 cm²; ~80 °C for ~14 s in one protocol) (Dolivo et al., 2023). Devices are applied to the skin under sustained pressure for a fixed exposure duration (often ~20 s) to target reproducible deep partial- to full-thickness injury (Henke et al., 2005; Shi et al., 2020; Mekkaoui et al., 2021; Anis et al., 2022). Post-procedurally, ketoprofen (e.g., 1 mg/kg) has been used for analgesia (Rahmani-Neishaboor et al., 2010). Given the severity of these injuries, multi-modal analgesia strategies combining opioids (e.g., buprenorphine) and NSAIDs (e.g., meloxicam) are increasingly recommended to ensure welfare and minimize pain-induced artifacts in data (Lussier et al., 2024).

Species selection: New Zealand White rabbits (including healthy male cohorts), typically 8–10 weeks old and weighing approximately 1,500–2,500 g (Henke et al., 2005; Shi et al., 2020; Mekkaoui et al., 2021).

Advantages: A commonly cited advantage of rabbit thermal wound models is the relatively limited contribution of contraction (often reported as <15%), with healing driven predominantly by granulation tissue formation and re-epithelialization, processes that better approximate human deep-burn repair than contraction-dominant dorsal rodent wounds (Mistry et al., 2022). This characteristic strengthens translational interpretability for endpoints such as epithelial coverage, granulation thickness/vascularity, and matrix remodeling. Rabbits provide sufficient body surface area to support multi-wound within-animal designs and repeated tissue sampling, enabling more robust time-course analyses than many murine paradigms (Henke et al., 2005; Shi et al., 2020; Mekkaoui et al., 2021). This is particularly relevant for burn studies where depth progression, inflammatory resolution, and scar remodeling unfold over extended periods.

Disadvantages: Although temperature and time are nominally specified, the delivered thermal dose is highly sensitive to contact pressure, device thermal mass, interface medium (gauze vs metal), and skin perfusion at the chosen site. “Constant pressure” is difficult to standardize without instrumentation, and small differences can shift burn depth. Consequently, claims of consistent grade II–III injury require cautious interpretation unless burn depth is confirmed histologically at early time points. Specifically, the zone of stasis—the area surrounding the central necrosis that is at risk of delayed infarction—is highly variable and sensitive to hydration status and infection, making it a critical but challenging target for therapeutic intervention. Open-flame heating of metal plates and transfer via boiled gauze may generate spatial temperature gradients and variable heat loss before skin contact. Without reporting plate surface temperature at contact and dwell-time precision, cross-study comparisons of “equivalent burns” are inherently limited (Henke et al., 2005; Shi et al., 2020; Mekkaoui et al., 2021; Anis et al., 2022).

Clinical relevance & biochemical validation: Rabbit thermal injury models have been reported to develop hypertrophic scar-like features with morphological similarity to human hypertrophic scars (Nabai and Ghahary, 2017a), supporting their use when the primary translational endpoint concerns long-term remodeling rather than acute closure alone. Moreover, the rabbit ear model, specifically when subjected to thermal injury, creates a unique environment where the cartilage prevents contraction, allowing for the precise measurement of hypertrophic scar height and volume, which correlates well with clinical scar scales.

Limitation: Immune, microbiome, & environment:

1. Regenerative potential: Rabbits possess a higher intrinsic regenerative capacity than humans (e.g., ear punch holes can regenerate cartilage). This biological “optimism” may lead to overestimating the regenerative efficacy of biomaterials.
2. Pain masking: As prey animals, rabbits mask signs of pain. This complicates the assessment of the “pain-stress-healing” loop, which is a major factor in human burn patients.

### 4.4 Porcine models: the translational gold standard

While rodent and rabbit models are invaluable for high-throughput screening, their predictive value for human clinical trials is limited by significant anatomical and physiological differences. Porcine skin is widely recognized as the closest surrogate to human skin, sharing striking similarities in epidermal thickness (70–140 μm), dermal-epidermal ridge structure, and hair follicle density (Seaton et al., 2015). Crucially, unlike rodents that heal primarily via contraction, pigs heal through re-epithelialization and granulation tissue formation—mechanisms identical to humans (Seaton et al., 2015).

Model description: The Red Duroc pig is particularly unique as it naturally forms thick, hyperpigmented scars that resemble human hypertrophic scars, making it the premier model for anti-scarring therapies (Liew et al., 2017). Wounds are typically created on the paravertebral dorsum or flank using dermatomes to ensure precise depth control (split-thickness vs. full-thickness).

Species selection: Common breeds include the domestic Yorkshire pig and the Red Duroc pig (Liew et al., 2017).

Advantages: Porcine skin is the closest surrogate to humans, sharing similar thickness and rete ridges. Crucially, pigs heal via re-epithelialization rather than rodent-like contraction, making them the gold standard for translational validity (Summerfield et al., 2015). The Red Duroc breed uniquely models human hypertrophic scarring, while large surface areas allow efficient split-body comparisons (Menchaca et al., 2023).

Disadvantages: High husbandry costs and facility requirements often limit sample sizes. Rapid somatic growth in piglets creates skin tension that can distort long-term scar assessments. Additionally, anesthetic risks like Malignant Hyperthermia and distinct skin microbiome differences (e.g., S. hyicus) complicate infection studies compared to human pathology (Lussier et al., 2024).

Clinical relevance & biochemical validation: The porcine model allows for the testing of standard human clinical dressings and devices (e.g., Negative Pressure Wound Therapy) without scaling issues. Furthermore, their immune system response—characterized by a similar timeline of neutrophil and macrophage infiltration—mirrors human inflammatory phases more accurately than murine systems (Gillum et al., 2022). Regulatory bodies such as the FDA increasingly mandate safety and efficacy data from porcine models before approving new wound care products for human trials. Recent advancements include the use of combustion-based burn models on pigs to test enzymatic debridement agents, a direct precursor to clinical use (Lindford et al., 2015). Porcine models exhibit a human-like profile of Matrix Metalloproteinases (MMPs) and Tissue Inhibitors of Metalloproteinases (TIMPs). Validating the restoration of the MMP/TIMP balance is crucial for chronic wound and burn studies. Additionally, the expression profiles of growth factors such as PDGF, VEGF, and FGF-2 in pig wounds closely mirror the temporal kinetics seen in human clinical samples (Smolle et al., 2017).

Limitation:

1. Rapid growth: Commercial piglets have an exponential growth rate. This rapid somatic growth applies “pre-tension” to the skin, which can alter scar mechanics and contraction forces compared to adult human skin.
2. Microbiome: While closer to humans than mice, the porcine skin microbiome (e.g., S. hyicus) differs from the human commensal flora (S. epidermidis), influencing biofilm formation dynamics.
3. Cost & ethics: High husbandry costs often limit sample sizes (n=3-5), potentially reducing statistical power compared to rodent studies.

### 4.5 Specialized injury models: electrical burns and contusion wounds

Beyond standard incision and thermal burn models, specific clinical etiologies require distinct modeling approaches to capture their unique pathophysiology.

#### 4.5.1 Electrical burn models

Electrical injuries are characterized by “progressive necrosis,” where tissue damage extends far beyond the initial contact site due to electroporation and vascular thrombosis. Rat Electrical Burn Model: A high-voltage (e.g., 1000–5000 V) current is applied to the dorsal skin of rats to simulate high-tension electrical burns. This model is critical for studying the “zone of stasis”—tissue that is initially viable but risks delayed necrosis—and for testing therapies that rescue perfusion, such as surfactant poloxamer-188 (Hao et al., 2024).Pathophysiological Relevance: Unlike thermal burns, electrical injury causes significant deep tissue (muscle and nerve) damage while sparing superficial skin layers in some areas, a phenomenon that standard thermal models fail to replicate.

#### 4.5.2 Contusion (crush) wound models

Contusion wounds, common in trauma and sports medicine, involve blunt force injury that disrupts soft tissue and microvasculature without necessarily breaching the skin surface initially.

Description: Controlled impact is delivered using a falling weight device (e.g., a metal rod dropped from a fixed height) onto the rodent dorsum or limb.

Significance: These models are essential for studying soft tissue edema, hematoma formation, and muscle regeneration. Recent studies utilize this model to evaluate the efficacy of photobiomodulation (PBM) and anti-inflammatory hydrogels in accelerating the resolution of deep tissue bruising and preventing secondary necrosis (Melo et al., 2011).

## 5 Chronic skin wound healing

### 5.1 Diabetes wound healing process

When the process of wound healing is disturbed by underlying pathological mechanisms or microbial invasion, for example, hyperglycemia, the wound fails to heal. It becomes chronic, as seen in diabetic wounds (Bi et al., 2022). Hyperglycemia impairs the synthesis, migration, and proliferation of proteins critical to re-epithelialization (Park et al., 2011; Andrade et al., 2017; Lima et al., 2017; Kim et al., 2018). In diabetic wounds, the expression of keratin-forming proteins involved in re-epithelialization is disrupted, particularly cytoskeletal keratins (K2, K6, and K10), which occupy a central position in keratinocyte differentiation. The precursor protein of the laminin-5α3 chain (LM-3A32) supports epithelial cell adhesion to the basement membrane (Blakytny and Jude, 2009). A decrease in LM-3A32 levels compromises keratinocyte survival and differentiation, thereby hindering the process of re-epithelialization (Ryan et al., 1999). Furthermore, the chronic wound environment is characterized by a persistent inflammatory state, where macrophages fail to switch from the pro-inflammatory M1 phenotype to the pro-reparative M2 phenotype. This stagnation leads to the excessive secretion of pro-inflammatory cytokines (e.g., TNF-α, IL-1β) and an imbalance between matrix metalloproteinases (MMPs) and their tissue inhibitors (TIMPs). The resulting overactivity of MMPs (particularly MMP-9) aggressively degrades the extracellular matrix and essential growth factors, preventing the formation of a stable scaffold for cell migration. Additionally, free radical damage caused by reduced activity of antioxidant enzymes, for example, glutathione peroxidase and superoxide dismutase, is another contributing factor (Dworzański et al., 2020). Hyperglycemia promotes the generation of reactive oxygen species (ROS) through pathways involving polyol, hexosamine, protein kinase C, and advanced glycation end-products (AGEs) (Deng et al., 2021). ROS are crucial for the early phases of wound healing (Rodriguez et al., 2008; Xu and Chisholm, 2014); however, excessive ROS production adversely affects later stages and prevents tissue from repairing. Elevated ROS levels specifically disrupt blood flow, metabolism, and peripheral nerve structures, resulting in sensory, motor, and autonomic dysfunction in affected nerves (Obrosova, 2003). This neuropathy, combined with microvascular dysfunction, reduces the patient’s ability to sense trauma and impedes the delivery of oxygen and nutrients to the wound site, creating a hypoxic environment that favors the formation of bacterial biofilms. In conclusion, elevated blood glucose levels increase the skin’s vulnerability to injury and infection, thereby delaying wound healing (Burgess et al., 2021).

### 5.2 Diabetic models

Animal diabetes models can be generated through chemical induction, dietary manipulation, surgical approaches, or genetic mutations, each producing distinct metabolic and inflammatory phenotypes (Bi et al., 2022). Chemical induction remains widely used because it is technically accessible and scalable. Among chemical agents, streptozotocin (STZ) is the most frequently adopted diabetogenic compound; it preferentially injures pancreatic β-cells, resulting in insulin deficiency with subsequent hyperglycemia and, in more severe settings, ketosis (Srinivasan and Ramarao, 2007). Importantly, the pathophysiological phenotype generated by STZ aligns most closely with type 1 diabetes–like insulinopenia, whereas type 2 diabetes–relevant features (obesity, insulin resistance, dyslipidemia) are incompletely represented unless STZ is combined with dietary induction or specific genetic backgrounds. To better model Type 2 diabetes, which accounts for the vast majority of clinical diabetic ulcer cases, researchers often utilize a High-Fat Diet (HFD) combined with low-dose STZ to simulate insulin resistance followed by β-cell dysfunction, or employ genetically modified strains such as Lepr^{db} (db/db) mice and Lepr^{ob} (ob/ob) mice that spontaneously develop severe obesity, hyperglycemia, and hyperinsulinemia. Therefore, in wound-healing studies, diabetes induction should be treated not merely as a prerequisite step but as a determinant of the inflammatory–metabolic milieu that drives delayed repair; the diabetic phenotype chosen should match the clinical context being modeled before wound creation (Bi et al., 2022).

#### 5.2.1 Diabetic wound models in mice

Model description: Mice are commonly used to establish diabetic wound models by inducing diabetes with intramuscular STZ administration, either as STZ alone or dissolved in 0.1 M sodium citrate buffer (pH 4.5).To reduce acute hypoglycemic stress immediately after STZ exposure, glucose water is provided for approximately 12 hours post-injection (Chen et al., 2020; Chen et al., 2023). Animals are typically maintained under controlled housing conditions (e.g., 23 ± 1 °C; 55% ± 5% humidity; 12 h light/dark cycle) with ad libitum food and water and a pre-experimental acclimation period (e.g., one week) (Wei et al., 2020). Reported criteria include (i) fasting blood glucose (FBG) ≥16.7 mmol/L sustained for at least one month (Leal et al., 2015), and (ii) FBG >11.5 mM measured as early as seven days post-STZ injection using a glucometer (Leal et al., 2015). While both thresholds are used in the literature, they represent materially different metabolic severities and stabilization times, which can translate into divergent wound phenotypes. This variability should be acknowledged explicitly in a review and, where possible, used to interpret between-study discrepancies. For wound creation, anesthesia is induced using intramuscular phenobarbital sodium (e.g., 0.3% solution, 0.1 mL/10 g) (Leal et al., 2015), pentobarbital sodium (e.g., 1%, 5 μL/g) (Xiang et al., 2024), or inhalational isoflurane (e.g., 2%) (Ban et al., 2020). After dorsal shaving and antisepsis (Leal et al., 2015), full-thickness excisional wounds (typically 5–10 mm in diameter) are generated with sterile biopsy punches/perforators (Park et al., 2015; Bi et al., 2022; Shi et al., 2022; Yuan et al., 2022). To minimize rodent-specific contraction and improve interpretability for epithelialization and granulation outcomes, silicone rings are sutured around the wound perimeter as splints (Park et al., 2015; Bi et al., 2022; Shi et al., 2022; Yuan et al., 2022).

Species selection: Male C57BL/Kunming mice (8–12 weeks old; 20–31 g) (Chen et al., 2020; Chen et al., 2023).

Advantages: The STZ diabetic mouse wound model is attractive for mechanistic studies because it is cost-efficient, scalable, and compatible with genetic manipulation and immunophenotyping. When paired with splinting, the excisional wound paradigm allows more meaningful interrogation of processes that are clinically relevant to diabetic wound delay—such as impaired re-epithelialization, attenuated angiogenesis, dysregulated macrophage polarization, and altered matrix remodeling—without the confounding dominance of contraction (Park et al., 2015; Bi et al., 2022; Shi et al., 2022; Yuan et al., 2022). In addition, the rapid induction timeline enables hypothesis testing and preliminary therapeutic screening within a manageable experimental window (Wei et al., 2020).

Disadvantages: STZ-induced diabetes predominantly models insulin-deficient disease and does not inherently reproduce the obesity/insulin-resistance milieu typical of many human diabetic ulcers; therefore, translational claims to type 2 diabetes should be framed cautiously unless additional metabolic features are incorporated (Srinivasan and Ramarao, 2007). STZ can introduce systemic stress beyond β-cell loss, and variations in dosing regimen, route, and supportive care (e.g., glucose supplementation) may influence inflammation, weight loss, and hydration status—factors that independently modulate wound healing trajectories. Furthermore, standard STZ models rarely spontaneously develop the macrovascular ischemia or significant peripheral neuropathy seen in human patients. To address this, some advanced protocols incorporate surgical ischemia (e.g., femoral artery ligation) or hindlimb unloading to better mimic the multifactorial etiology of diabetic foot ulcers. The wide range of diagnostic thresholds (FBG >11.5 mM at day 7 vs ≥16.7 mmol/L sustained for ≥1 month) reflects differences in disease severity and stabilization, which can produce inconsistent wound outcomes and complicate synthesis across studies (Leal et al., 2015). Even with splinting, mice differ from humans in skin appendage density, immune kinetics, and angiogenic responses; these interspecies differences can shift the relative contribution of inflammatory resolution versus vascular impairment in driving delayed repair (Martin, 1997). Small body size limits repeated large-volume sampling and can constrain certain delivery strategies or longitudinal biomaterial testing compared with larger species.

Clinical relevance & biochemical validation: Overall, STZ-induced diabetic mouse wounds—particularly when splinted—are best positioned for molecular and cellular mechanism discovery, gene-function interrogation, and early-stage screening of candidate interventions targeting impaired angiogenesis, inflammation resolution, or matrix remodeling in an insulin-deficient context (Chen et al., 2020; Chen et al., 2023). However, given the high prevalence of infection in clinical diabetic wounds, increasing translational value can be achieved by inoculating these wounds with biofilm-forming bacteria (e.g., *S. aureus*), thereby creating a composite model of diabetes, ischemia, and infection. For claims centered on chronic, ischemic, neuropathic, or obesity-associated diabetic ulcers, this model should be treated as a partial analogue and ideally complemented by models that capture the relevant comorbid drivers (e.g., insulin resistance, peripheral ischemia, neuropathy) (Bi et al., 2022). In the spectrum of diabetic wound models, STZ-induced splinted excisional wounds in mice provide a high-throughput platform for mechanistic discovery in an insulin-deficient milieu, but their translational scope is constrained for type 2 diabetes–dominant clinical ulcers unless obesity/insulin resistance and chronic comorbidities are explicitly modeled (Chen et al., 2023). This model biochemically replicates the “Chronic Wound Microenvironment”. Analysis consistently shows High Protease Activity (MMP-9) which degrades growth factors, and Low Growth Factor levels (IGF-1, TGF-β). Critically, it validates the accumulation of Advanced Glycation End-products (AGEs) and the activation of their receptor (RAGE), which perpetuates NF-κB mediated inflammation. It also shows defective Macrophage efferocytosis (clearance of apoptotic cells), a key cellular mechanism of chronicity (Shi et al., 2022).

Limitations: Translational gaps: immune, microbiome, & environment:

1. Metabolic discrepancy: db/db mice are morbidly obese but do not develop the macrovascular atherosclerosis (plaque rupture) typical of human diabetics. They model the metabolic toxicity (glucotoxicity) but not the ischemic infarct pathology of the human diabetic foot.
2. Microbiome shift: The diabetic skin microbiome in humans shifts dramatically toward pathogenic anaerobes. In SPF db/db mice, this shift is absent unless artificially induced. Thus, the model misses the “polymicrobial synergy” that drives non-healing in humans.
3. Age & epigenetics: Diabetes in humans is a decades-long disease causing epigenetic changes (hyperglycemic memory). db/db mice develop diabetes rapidly (4–8 weeks). They lack the long-term epigenetic chromatin modifications on inflammatory genes that make human diabetic wounds so refractory to treatment.

#### 5.2.2 Diabetic wound models in rats

Model description: For chemical induction, rats receive intraperitoneal STZ (e.g., 65 mg/kg) diluted in citrate buffer (0.1 M; pH ~4) to generate a predominantly type 1 diabetes–like insulin-deficient phenotype (Chung et al., 2022). Glycemic confirmation is frequently based on non-fasting glucose measurements (e.g., ≥250 mg/dL) within the first week following STZ administration using a glucometer (Accu-Chek) (Keshk et al., 2020).

Critical note: a one-week confirmation window can document hyperglycemia but may not guarantee metabolic stabilization; disease severity and systemic catabolism can evolve over subsequent weeks, potentially altering wound outcomes. In a review, it is important to acknowledge that thresholds based on non-fasting glucose and short post-induction windows can introduce between-study heterogeneity and complicate comparisons across protocols. Anesthesia is achieved via intramuscular ketamine combined with toluene thiazide (reported as 20 mg/kg and 4 mg/kg, respectively) (Keshk et al., 2020) or via inhalational isoflurane (e.g., 2%) (Cheng et al., 2022). After dorsal hair removal and antisepsis (e.g., 10% povidone-iodine) (Binsuwaidan et al., 2022), full-thickness excisional wounds are created on the dorsum, commonly as square defects with an area of approximately 1.5–3.0 cm² using scalpels or surgical scissors (Lindford et al., 2015; Menchaca et al., 2023). The day of wounding is defined as day 0 (Keshk et al., 2020). Postoperative management in the cited protocols includes antisepsis and analgesia (e.g., paracetamol administered via drinking water) (Cheng et al., 2022; Chung et al., 2022).

Species selection: Male Wistar albino, female Wistar, and ZDSD rats (7–12 weeks; 185–250 g ± 30 g) (Keshk et al., 2020; Zhang et al., 2021; Chung et al., 2022).

Advantages: Relative to mice, rat models offer a larger operative field that facilitates standardized wound creation, repeated sampling, and clinically relevant interventions (e.g., dressing changes, topical delivery, local injections). Rat skin and metabolic physiology are frequently regarded as closer to humans than murine systems, which can improve the interpretability of endpoints such as granulation tissue development and angiogenesis (Avci et al., 2013; Kleinert et al., 2018). These features support medium-scale efficacy testing of wound dressings and therapeutics and enable more informative longitudinal analyses than many mouse workflows (Mistry et al., 2022). Furthermore, the larger body size of rats permits the incorporation of surgical refinements to mimic diabetic complications more accurately. For instance, creating hindlimb ischemia via femoral artery ligation in diabetic rats reproduces the peripheral arterial disease (PAD) component of human diabetic foot ulcers, allowing researchers to study wound healing under conditions of both hyperglycemia and macrovascular insufficiency.

Disadvantages: As with other rodent cutaneous injuries, dorsal excisional closure can be substantially influenced by contraction, which may overstate “healing” if closure rate is the primary endpoint. Where translational alignment is essential, contraction-limiting approaches (e.g., splinting) should be considered or, at minimum, contraction should be quantified and reported. STZ protocols model insulin deficiency rather than the insulin resistance/obesity milieu typical of many chronic diabetic ulcers; moreover, STZ-induced systemic illness and weight loss can independently influence inflammation and tissue repair, complicating mechanistic attribution. Reliance on non-fasting glucose ≥250 mg/dL within one week may capture early hyperglycemia but can miss later stabilization variability (Keshk et al., 2020). Routine use of povidone-iodine antisepsis and paracetamol analgesia is ethically appropriate, yet both can modulate local inflammation or microbial burden and thereby shift wound-healing trajectories—an important consideration when interpreting cytokine endpoints or infection-prone diabetic wounds (Cheng et al., 2022). Specifically, povidone-iodine is cytotoxic to fibroblasts and keratinocytes at certain concentrations, which may inadvertently delay healing and confound the assessment of regenerative therapies. Species-specific immune cell functions and cytokine expression patterns can limit direct translation to human diabetic ulcers, particularly for immunomodulatory interventions (Werner and Grose, 2003).

Clinical relevance & biochemical validation: Zucker Diabetic–Sprague Dawley (ZDSD) rats provide an alternative platform with broader metabolic features more consistent with type 2 diabetes–like disease (Keshk et al., 2020; Zhang et al., 2021; Chung et al., 2022). This distinction is not merely taxonomic: the dominant drivers of delayed repair differ between insulinopenia and insulin resistance/obesity-associated inflammation, and model choice should therefore be explicitly aligned to the clinical question. This model validates the Oxidative Stress theory of diabetic complications (Deng et al., 2021). Wound tissue analysis reveals elevated Reactive Oxygen Species (ROS) and Nitrotyrosine, leading to the uncoupling of eNOS and impaired NO production. This biochemical defect directly impairs endothelial cell function (Catrina and Zheng, 2016). The model is used to test Nrf2 pathway activators (antioxidants) by measuring downstream HO-1 and SOD expression (Xiang et al., 2024). Rat diabetic wound models are particularly suitable for studies emphasizing (i) dynamic assessment of wound repair under hyperglycemia, (ii) evaluation of dressings/biomaterials and locally delivered therapeutics, and (iii) mechanistic links between metabolic state and angiogenesis/granulation outcomes (Avci et al., 2013; Kleinert et al., 2018). (iv) Evaluation of antimicrobial efficacy in chronic wounds: Rats are robust enough to support infected wound models where diabetic ulcers are inoculated with biofilm-forming bacteria (e.g., Pseudomonas aeruginosa or methicillin-resistant *Staphylococcus aureus* [MRSA]), providing a crucial platform for testing dressings intended for infected diabetic foot ulcers. For clinical claims centered on type 2 diabetes–dominant ulcers, metabolically relevant strains such as ZDSD may offer improved construct validity compared with STZ alone, while STZ models remain useful for insulinopenic phenotypes and for probing glucose toxicity–linked repair defects (Chung et al., 2022). Overall, rat diabetic wound models provide greater procedural flexibility and sampling depth than mice, but their translational value depends on matching the diabetic phenotype (STZ-induced insulinopenia versus metabolically driven strains such as ZDSD) to the intended clinical context and on accounting for contraction bias and perioperative medication effects when interpreting wound endpoints (Mistry et al., 2022).

Limitations: Translational gaps: immune, microbiome, & environment:

1. Collateral circulation: Rats have extensive collateral blood vessel networks. Femoral ligation produces transient ischemia that resolves in days, whereas human critical limb ischemia (CLI) is chronic and progressive. This makes the rat model a “best case scenario” rather than a true mimic of the “worst case” human CLI.
2. Neuropathy: While metabolic changes occur, rats do not spontaneously develop the profound peripheral sensory neuropathy (Charcot foot) that causes mechanical overloading and ulceration in humans. The “loss of protective sensation” component is missing.

#### 5.2.3 Diabetic wound models in rabbits

Model description: Two major induction strategies are reported: (1) STZ-based induction: intramuscular administration of STZ (e.g., 50 mg/kg in 0.1 mol/L citrate buffer) once daily for five consecutive days (Pradhan et al., 2011; Pradhan Nabzdyk et al., 2013). (2) Alloxan-based induction: intravenous administration of alloxan monohydrate (e.g., 75 mg/kg) (Pradhan Nabzdyk et al., 2013) or alloxan prepared in saline and infused via an ear vein cannula at a controlled rate (e.g., 1.5 mL/min) (O’Loughlin et al., 2013b). Diabetes is commonly considered established when blood glucose reaches ≥250 mg/dL (O’Loughlin et al., 2013b). while this threshold is convenient and widely used, a single glucose cutoff does not fully characterize the metabolic phenotype relevant to chronic diabetic wound pathology (e.g., stability over time, weight trajectory, hydration status, and insulin levels). In a review, it is therefore important to distinguish “hyperglycemia achieved” from “diabetic state stabilized,” because variability in stabilization time and systemic catabolism can materially influence wound outcomes and intervention readouts. After confirmation of hyperglycemia, rabbits are anesthetized using intramuscular ketamine (reported across a wide dose range, e.g., 100–120 mg/kg) (O’Loughlin et al., 2013b; Fatima et al., 2022; Elshazly et al., 2023), with adjunct agents such as toluene thiazide (e.g., 10 mg/kg) (Pradhan et al., 2011; Pradhan Nabzdyk et al., 2013) or xylazine (e.g., 0.1 mL/kg) (Leal et al., 2015) depending on protocol. Following shaving and antisepsis, full-thickness circular wounds are commonly created using sterile biopsy punches (e.g., 6 mm) on the leg or ear skin (Leal et al., 2015). Postoperative care may include antibiotic prophylaxis (e.g., enrofloxacin 5 mg/kg) and opioid-based analgesia (O’Loughlin et al., 2013a). although clinically reasonable, routine antibiotics and opioids can alter microbial burden and inflammatory tone; this should be acknowledged when comparing cytokine endpoints, macrophage phenotypes, or infection-related outcomes across studies.

Species selection: New Zealand White or Himalayan rabbits (approximately 3–12 months old; 2.5–3.5 kg) using diabetogenic agents that primarily target pancreatic β-cells (Pradhan et al., 2011; Pradhan Nabzdyk et al., 2013).

Advantages: Rabbits offer a cutaneous architecture and thickness that can be closer to humans than small rodents, and their remodeling/scarring trajectories are often considered more clinically informative for translational wound research (Mistry et al., 2022). This can enhance interpretability for endpoints such as granulation quality, re-epithelialization kinetics, and scar formation under hyperglycemic conditions—particularly when the study aim extends beyond early inflammatory signaling to longer-term remodeling. The larger body size enables extended follow-up, more complex local interventions (e.g., advanced dressings, staged procedures), and repeated sampling with less technical constraint than in mice (Pradhan et al., 2011). This feature is especially valuable when evaluating material-based therapies or delivery systems that require consistent placement, dressing retention, or serial tissue collection. A unique and highly relevant modification in rabbits is the ischemic ear ulcer model (often referred to as Stryker’s model). By surgically interrupting the central and marginal arteries of the rabbit ear while leaving the skin intact, researchers can create a reproducible ischemic zone. When combined with diabetes induction, this model uniquely recapitulates the neuro-ischemic nature of human diabetic foot ulcers, providing a rigorous testbed for therapies targeting angiogenesis and oxygenation.

Disadvantages: Both STZ and alloxan are β-cell toxins; therefore, rabbit models induced by these agents primarily resemble insulin-deficient diabetes, which may not fully capture the insulin resistance/obesity-associated milieu typical of many human diabetic foot ulcers. Translational claims to type 2 diabetes–dominant clinical scenarios should be framed cautiously unless additional metabolic features are modeled or clearly characterized. High inter-individual variability and induction risk. Rabbits can show substantial variability in tolerance to STZ/alloxan, which can reduce induction success rate, increase morbidity, and compromise model stability—an important practical constraint for reproducibility and sample size planning (Obrosova, 2003; Rodriguez et al., 2008; Xu and Chisholm, 2014). Anatomical site heterogeneity (ear vs leg). Wound location is not a neutral choice: ear skin differs from leg skin in thickness, vascularity, mechanical environment, and adjacency to underlying structures, all of which can influence epithelialization and scarring. Therefore, outcomes from ear versus leg wounds should not be pooled without accounting for site effects. Cost and throughput. High husbandry cost and slower reproduction limit large-scale screening; rabbits are better positioned as a translational validation platform rather than a discovery-stage workhorse.

Clinical relevance & biochemical validation: In summary, rabbit diabetic wound models provide a higher-fidelity cutaneous platform for evaluating morphological and functional repair under hyperglycemia and for testing complex local interventions that are difficult to implement in smaller rodents (Mistry et al., 2022). Their greatest value lies in late-phase remodeling and scar-oriented endpoints and in preclinical validation before clinical translation. However, interpretability depends on (i) careful characterization and stabilization of the diabetic phenotype beyond a single glucose threshold, (ii) explicit reporting of wound site and perioperative medications, and (iii) recognition that STZ/alloxan induction primarily captures insulin-deficient diabetes rather than the full spectrum of human diabetic ulcer pathophysiology (Obrosova, 2003; Rodriguez et al., 2008; Xu and Chisholm, 2014). Compared with rodent diabetic wound models, rabbits enable longer follow-up and clinically relevant local interventions with more human-like skin remodeling, but their β-cell toxin–based induction strategies, inter-individual variability, and cost constrain throughput and require cautious phenotype characterization for robust translation (Obrosova, 2003; Rodriguez et al., 2008; Xu and Chisholm, 2014). Finally, while rabbit models offer significant advantages, therapies showing promise here should ideally be confirmed in porcine diabetic models if possible, as pig skin’s anatomical and physiological similarity to human skin remains the gold standard for predicting clinical efficacy (Seaton et al., 2015). This model validates the loss of Neuro-cutaneous Signaling. Biochemical analysis shows a depletion of neuropeptides like Substance P and CGRP, which are crucial for initiating the inflammatory flare. It serves as a rigorous platform for testing therapies that aim to restore HIF-1α stability and neovascularization under conditions of severe hypoxia and glucose toxicity (Catrina and Zheng, 2016).

Limitations: Translational gaps: immune, microbiome, & environment:

1. Fragility: The induction of diabetes in rabbits (via Alloxan) is toxic and creates a systemic state of “critical illness” rather than stable chronic diabetes. The high mortality and systemic catabolism can confound wound healing data—are the wounds slow to heal because of “diabetes” or because the animal is dying of multiple organ failure?
2. Housing stress: Similar to other rabbit models, the stress of single-housing and daily glucose checks (ear pricks) induces a stress response that can skew immune function, a factor rarely controlled for in comparison to human patients living in varied social environments.

## 6 Pathological scar models

### 6.1 Tension-induced scar model

The tension-induced scar model is a type of experimental model based on the pathophysiological mechanism that “mechanical tension is a core inducer of scar hyperplasia (Gurtner et al., 2008).” It simulates the tension microenvironment during clinical wound healing by applying controllable mechanical stress in experimental animals or *in vitro* systems, thereby inducing the formation of hypertrophic scar-like tissue. The core value of this model lies in addressing the limitation of traditional scar models that ignore mechanical factors—clinical studies have confirmed that the incidence of hypertrophic scars in areas with sustained tension (such as joints, chest, and shoulders) is 3–5 times higher than in tension-free areas (Mascharak et al., 2021). By accurately reproducing this key pathological factor, such models provide a clinically relevant experimental vehicle for elucidating the molecular mechanisms of tension-regulated scar formation and developing anti-scar intervention strategies. Their application scenarios cover basic mechanism research, drug screening, physical therapy optimization, and other fields, making them an indispensable core tool in scar research, particularly serving as a bridge connecting basic research and clinical translation (He desJardins et al., 2020).

#### 6.1.1 Mice scar model

Model description: A linear incision is made on the back of mice, and the upper edge of the incision is sutured and fixed to the rib cage to apply continuous directional tension, with the lower edge serving as a tension-free control. Hypertrophic scars are induced by inhibiting wound contraction. The model can stably maintain the scar phenotype for at least 60 days, and pathological examination reveals cell proliferation, dense blood vessels, and disordered collagen deposition (Byrne and Aly, 2019).

Species selection: C57BL/6 mice, particularly Piezo1-knockout or YAP/TAZ transgenic lines.

Advantages: Allows for the isolation of Mechanical Force as an independent variable. Recent innovations include wireless, bioresorbable sensors that monitor wound strain and stiffness in real-time.

Disadvantages: Device dislodgement; foreign body reaction.

Clinical relevance & biochemical validation: This model is the definitive tool for Mechanotransduction research. It biochemically validates the Hippo Signaling Pathway: mechanical stress triggers the nuclear translocation of YAP and TAZ, which bind to TEAD transcription factors to upregulate profibrotic genes (CTGF, Cyr61) (Lv et al., 2024). It also confirms the role of the mechanosensitive ion channel Piezo1, linking physical stretch to intracellular Calcium influx and fibrosis (Zhao et al., 2026).

Limitations: Mouse skin often stretches (“creep”) rather than forming a true keloid-like scar.

#### 6.1.2 Rabbit ear hypertrophic scar Model

Model description: Four to six 6-mm excisional wounds are made on the ventral ear, ensuring the perichondrium is removed. The wounds heal under the natural tension of the skin-cartilage interface. The lack of contraction and delayed epithelialization leads to a raised, erythematous scar that persists for >6 months. Some protocols use Bleomycin injections to further exacerbate the fibrosis (Yang et al., 2023).

Species selection: New Zealand White rabbits. The only standard lab animal that naturally forms hypertrophic scars.

Advantages: Industry standard for anti-scarring drug screens. It exhibits the specific histological architecture of human scars (collagen nodules).

Disadvantages: Does not invade surrounding tissue like human Keloids.

Clinical relevance & biochemical validation: This model validates the Canonical TGF-β/Smad Pathway. Biochemical analysis consistently reveals elevated TGF-β1 levels and a high ratio of TIMP-1/MMP-1, indicating reduced collagen degradation. It is also used to measure Heat Shock Protein 47 (HSP47), a collagen chaperone. Therapies that successfully reduce the Scar Elevation Index (SEI) in this model almost always show a corresponding reduction in these biochemical markers (Liu et al., 2025).

Limitations: Anatomical differences (no fat) compared to body scars.

## 7 Sex as a biological variable: impact on wound healing and modeling bias

Historically, preclinical wound healing research has been predominantly conducted in male animals. This bias stems from the desire to minimize experimental variability associated with the female estrous cycle. However, this “male-centric” approach ignores a fundamental biological reality: wound healing is sexually dimorphic (Kearney et al., 2022). Clinical data reveal that chronic venous leg ulcers are more prevalent in women (particularly post-menopausal), while males often exhibit delayed acute wound healing responses. Failing to account for sex as a biological variable (SABV) creates a translational blind spot, potentially obscuring therapeutic efficacy or safety signals relevant to female patients (Klein and Flanagan, 2016).

### 7.1 Hormonal regulation of inflammation and angiogenesis

Sex steroids, particularly estrogens and androgens, act as potent modulators of the molecular phases of repair.

- Estrogen (Protective Role): Estrogens (primarily 17β-estradiol) generally accelerate cutaneous wound healing. They dampen the excessive inflammatory response by inhibiting the infiltration of neutrophils and suppressing the release of pro-inflammatory cytokines (e.g., TNF-α, macrophage migration inhibitory factor) (Keshk et al., 2020). Furthermore, estrogen promotes the polarization of macrophages toward the reparative M2 phenotype and directly stimulates angiogenesis by upregulating VEGF expression in endothelial cells (Horng et al., 2017). In ovariectomized (OVX) animal models—which simulate the post-menopausal state—wound healing is significantly delayed, characterized by reduced collagen deposition and excessive elastase activity, a phenotype reversible with topical estrogen treatment (Wen et al., 2022).
- Androgen (Deleterious Role): Conversely, androgens (testosterone and dihydrotestosterone) act as negative regulators of repair. They have been shown to sustain the inflammatory phase by enhancing TNF-α production and delaying re-epithelialization. Castration in male mice accelerates wound closure to rates comparable to females, suggesting that endogenous androgens contribute to the slower healing observed in males (Larouche et al., 2018).

### 7.2 The consequence of bias in current models

The systematic exclusion of female animals, or the failure to analyze data by sex, compromises external validity. For instance, testing an anti-inflammatory drug solely in male mice (which have a naturally higher inflammatory baseline due to androgens) may overestimate the drug’s efficacy compared to a female cohort. Conversely, using young, cycling female mice to model diabetic ulcers intended for an elderly, post-menopausal demographic introduces a “protection bias,” as their high estrogen levels may mask the severity of diabetic impairment (Horng et al., 2017).

### 7.3 Strategic recommendations for future experimental design

To improve the translational rigor of wound healing research, we propose the following experimental standards: Adopt SABV (Sex as a Biological Variable): Initial screening studies should include both male and female animals. Data should be disaggregated by sex to detect dimorphic effects of therapeutic interventions (Horng et al., 2017).

Match the Hormone Status to the Clinical Indication:

- For General Acute Wounds: Use both sexes to ensure safety and efficacy across the population.
- For Chronic Venous/Diabetic Ulcers in Elderly: Utilize Ovariectomized (OVX) female rats or mice (combined with diabetes/aging) to recapitulate the estrogen-depleted microenvironment of post-menopausal patients (Deng et al., 2021).
- For Hypertrophic Scarring: Given that investigating sex differences in fibrosis is emerging, verify if the chosen model (e.g., rabbit ear) exhibits sex-dependent collagen deposition rates.

Report Estrous Phase: When using female rodents, simply recording the estrous stage (via vaginal cytology) or randomizing across stages can mathematically account for variability without necessitating the exclusion of females (Beery, 2018).

## 8 Critical biological and environmental constraints in translational modeling

While anatomical differences like the panniculus carnosus are widely acknowledged, several systemic physiological factors critically limit the external validity of animal models. These factors—often overlooked in standard study designs—significantly contribute to the failure of clinical translation.

### 8.1 Immunological divergence

The immune system orchestrates the transition from inflammation to proliferation, yet profound differences exist between species. Murine blood is lymphocyte-rich (75–90%), whereas human blood is neutrophil-rich (50–70%), fundamentally altering the initial inflammatory surge post-injury (Zhang et al., 2022). Furthermore, laboratory mice housed in Specific Pathogen-Free (SPF) conditions possess a “naive” immune system akin to that of human neonates, lacking the memory T-cell maturity driven by environmental exposure in adult humans (Masopust et al., 2017). Consequently, rodent models often exhibit a more robust and resolved regenerative response, failing to recapitulate the chronic, low-grade, non-resolving inflammation characteristic of human diabetic ulcers.

### 8.2 The cutaneous microbiome gap

Human skin is colonized by a diverse “wild” microbiota that educates the local immune system. In contrast, standard laboratory animals are maintained in ultra-hygienic SPF environments with a restricted, uniform microbiome. This artificial sterility limits the model’s ability to simulate the polymicrobial biofilms (e.g., *S. aureus* and P. aeruginosa co-infections) that perpetuate chronicity in human wounds (Kalan et al., 2019). Evidence suggests that transferring “wild” microbiota to laboratory mice can shift their immune profile closer to humans, offering a potential strategy to enhance translational relevance (Rosshart et al., 2017).

### 8.3 Age and senescence

A striking paradox in wound healing research is the demographic mismatch: human chronic wounds predominantly afflict the elderly, yet preclinical studies overwhelmingly utilize young, healthy animals (8–12 weeks old). Young animals possess robust stem cell populations and efficient clearance of senescent cells. In contrast, elderly human tissues accumulate senescent fibroblasts and keratinocytes that secrete a Senescence-Associated Secretory Phenotype (SASP), driving persistent inflammation and degrading the ECM (Wilkinson and Hardman, 2020). Models that fail to use aged animals (e.g., >18 months for mice) essentially model “juvenile repair” rather than the geriatric pathology of non-healing ulcers (Ellis et al., 2018).

### 8.4 Environmental stress (housing temperature)

Physiological stress caused by standard housing temperatures introduces a hidden variable. Mice are typically housed at 20–22 °C, which is well below their thermoneutral zone (approx. 30 °C). Under this mild cold stress, mice divert significant metabolic energy toward thermogenesis via norepinephrine release, which induces vasoconstriction and suppresses the immune response (Hylander and Repasky, 2016). Recent studies have demonstrated that mice housed at thermoneutrality exhibit immune kinetics and healing rates that more closely mirror human physiology (Stemmer et al., 2015). Ignoring this variable can lead to an underestimation of the inflammatory phase and altered drug pharmacokinetics.

## 9 Strategic guide for model selection

**Figure 3:**
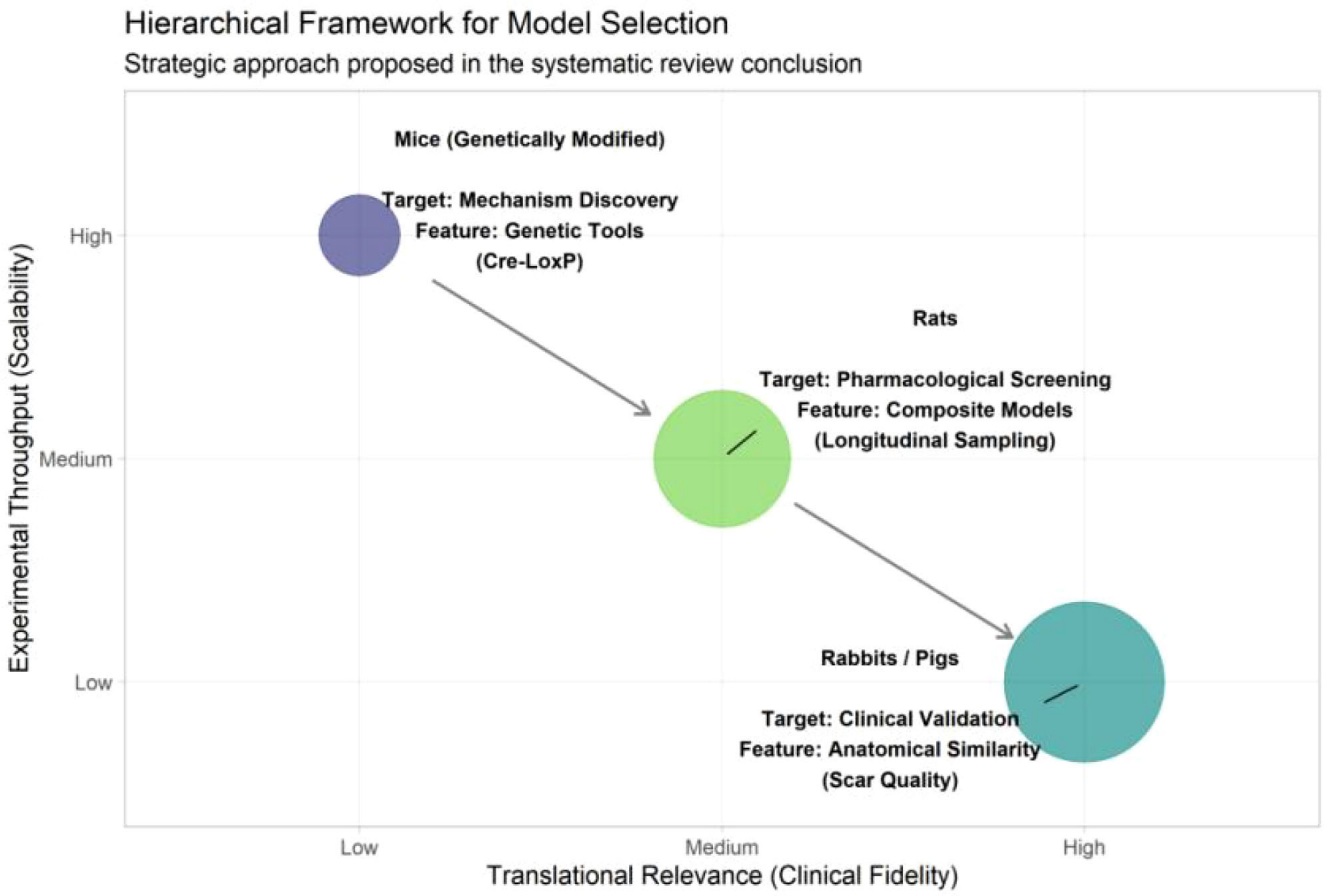
Stratification of animal models based on experimental scalability versus translational relevance.

Based on the systematic review of 129 studies and the critical appraisal of physiological discrepancies, we propose a hierarchical framework (**Figure 3**; **Tables 1**, **2**) to guide model selection according to clinical indications:

**Table 1:** Summarizes cross-species comparisons.

Species	Diabetes induction method	Wound establishment & healing mechanism	Recommended critical endpoints (beyond closure rate)	Advantages	Critical limitations	Translational relevance & Use case
Mouse	STZ (low-dose, multiple) or genetic (db/db, ob/ob)	Dorsal splinted excisional wounds. Healing Mechanism: Primarily contraction, even with splinting. Bio-deviation: Loose skin with active panniculus carnosus muscle layer.	Histology (re-epithelialization, granulation); Immunohistochemistry (angiogenesis, inflammation markers like F4/80); Cellular analysis (FACS).	Low cost; High throughput; Extensive genetic tools available (transgenic/knockout strains).	Contraction significantly overestimates true re-epithelialization. STZ models insulin deficiency (Type 1), not resistance (Type 2). High metabolic rate. Common sex bias (mostly males).	Early-stage mechanism discovery. Ideal for dissecting specific genetic or immune pathways (e.g., macrophage polarization, growth factor signaling).
Rat	STZ (single high-dose) or genetic (ZDF, ZDSD)	Dorsal excisional wounds. Healing Mechanism: Dominated by contraction. Bio-deviation: Presence of panniculus carnosus, though less active than in mice.	Tensile strength testing; Collagen analysis (Sirius Red, Second-harmonic generation); Serial blood/tissue sampling for systemic markers.	Larger tissue/blood volume than mice allows for more complex sampling; Some strains (ZDSD) better model Type 2 diabetes.	Contraction remains a major confounding factor. STZ induces significant systemic stress and off-target toxicity. Common sex bias.	Pharmacological screening. Good for preliminary testing of topical drugs, dressings, and biomaterials before moving to larger models.
Rabbit	Alloxan or STZ (multiple high-doses)	Full-thickness ear wounds. Healing Mechanism: Primarily re-epithelialization (skin is adherent to cartilage). Bio-deviation: Ear model lacks panniculus carnosus, but cartilage microenvironment is unique.	Scar assessment (hypertrophy, elevation index); Long-term collagen organization; Histological scoring of fibrosis and inflammation.	Naturally forms hypertrophic scars, similar to humans. Allows for long-term (>100 days) studies of scar maturation.	High inter-individual variability in glycemic control and wound healing; More expensive and difficult to handle than rodents.	Fibrosis and scar-related research. Preclinical validation of anti-scarring therapies; Investigating the transition from healing to pathological fibrosis.
Porcine (Pig)	High-fat diet + low-dose STZ; or genetic models	Full-thickness dorsal excisional wounds. Healing Mechanism: Re-epithelialization, very similar to humans. Bio-deviation: Closest to human: Similar epidermal/dermal thickness, hair follicle density, and dermal vasculature. No panniculus carnosus.	All human-like endpoints: Planimetry for re-epithelialization %; Biomechanical testing; Pigmentation and cosmetic outcome; Long-term biopsy for scar remodeling.	Gold standard for translational relevance. Large wound size allows for testing of human-scale devices and dressings. Wound progression mimics human chronic ulcers.	High cost and resource-intensive; Significant ethical considerations and regulatory oversight; Rapid growth of young animals can be a confounding variable.	Pivotal preclinical validation. Essential for generating robust data for regulatory submissions (e.g., FDA). Testing advanced therapies, wound-closure devices, and combination treatments.

This table summarizes the distinct characteristics of murine, leporine, and porcine models, contrasting their diabetes induction protocols and dominant wound repair mechanisms (rodent-mediated contraction vs. human-like re-epithelialization). It outlines critical bio-deviations, such as the presence of the panniculus carnosus, and recommends specific endpoints for assessing efficacy. Strategic guidance is provided on the translational relevance of each species, categorizing their utility from early-stage mechanistic discovery to pivotal preclinical validation for regulatory submission.

STZ, streptozotocin; ZDF, Zucker Diabetic Fatty; ZDSD, Zucker Diabetic Sprague Dawley; FACS, fluorescence-activated cell sorting.

**Table 2:** Comparative analysis of biological characteristics, healing mechanisms, and translational utility across key animal models.

Species	Model characteristics & biological deviations	Dominant healing mechanism	Recommended critical endpoints	Translational pros & cons	Best translational application
Mouse	• Loose skin with panniculus carnosus muscle layer. • Sparse hair follicles; thin epidermis. • Genetic tools (Cre-LoxP) are unmatched. • High metabolic rate.	Contraction (rapid, ~80-90% of closure) *Unless splinted	• Histology: Re-epithelialization length (gap). • Molecular: PCR/RNA-seq for pathways. • Cellular: FACS for immune profiling.	Pros: Low cost; High throughput; Best for genetic mechanism discovery. Cons: Contraction overestimates healing; Immune system is ‘naive’ (SPF).	Early-stage mechanism discovery (e.g., macrophage polarization, cytokine pathways).
Rat	• Loose skin with panniculus carnosus. • Larger body surface allows serial blood sampling. • No spontaneous hypertrophic scarring. • Often lacks sex-dimorphism in study designs.	Contraction (dominant in dorsal wounds) *Unless splinted	• Biomechanical: Tensile strength. • Histology: Collagen maturity (Type I/III ratio). • Systemic: Serum biomarkers (oxidative stress).	Pros: Supports complex surgery (flaps); Tissue volume allows multi-omics; Good for toxicology. Cons: Higher cost; Contraction bias persists; Infection models are forced (not chronic).	Pharmacological screening; Ischemia-reperfusion models; Composite tissue healing.
Rabbit	• Ear skin: Tightly adherent to cartilage (No panniculus effect). • Dorsal skin: Still loose. • High density of sebaceous glands. • Naturally forms hypertrophic scars (Ear model).	Re-epithelialization (Ear model) Contraction (Dorsal model)	• Scarring: Scar Elevation Index (SEI). • Morphology: Collagen bundle alignment. • Fibrosis: α-SMA expression.	Pros: Gold standard for anti-scarring drugs; Paired design (internal control); Human-like scar histology. Cons: High cost; Stress-sensitive; Ear cartilage differs from body skin.	Pathological scarring (Keloid/Hypertrophic) research; Anti-fibrotic biomaterial testing.
Pig	• Structurally closest to human (70–140 μm epidermal thickness). • Fixed skin structure (No panniculus in flank). • Similar hair follicle density and dermal collagen. • Rapid somatic growth.	Re-epithelialization & Granulation (Highly homologous to humans)	• Clinical Proxies: Visual Scar Scales (VSS). • Long-term: Pigmentation & remodeling. • Efficacy: Device performance testing.	Pros: FDA preferred for IND; Allows human-sized dressings; Gold standard for translation. Cons: Prohibitive cost; Ethical/Logistical complexity; Rapid growth affects long-term data.	Final pre-clinical validation (Safety/Efficacy); Testing medical devices & advanced dressings.

IND, Investigational New Drug application; SPF, Specific Pathogen Free; PCR, Polymerase Chain Reaction; FACS, Fluorescence-activated Cell Sorting.

This table synthesizes the physiological deviations, dominant closure mechanisms, and recommended experimental endpoints for the four primary species used in wound healing research. “Dominant Healing Mechanism” highlights the critical distinction between contraction-mediated closure (rodents) and re-epithelialization (human-like). “Best Translational Application” provides a hierarchical guide for model selection based on the specific clinical indication (e.g., scarring vs. chronic ulceration). Abbreviations: IND, Investigational New Drug application; SPF, Specific Pathogen Free; SEI, Scar Elevation Index; α-SMA, alpha-smooth muscle actin.

### 9.1 For chronic ulcers (diabetic/venous)

Do not use: Simple acute excision in healthy mice (poor predictive value).

Recommended: db/db mice with splinting (to enforce re-epithelialization) or rabbit ear ischemic models.

Critical validation: Efficacy must be confirmed by assessing re-epithelialization quality (not just rate) and angiogenesis in an ischemic microenvironment.

### 9.2 For pathological scarring (hypertrophic scars/keloids)

Do not use: Dorsal rat/mouse incision models (tension-free healing leads to fine scars).

Recommended: Rabbit ear hypertrophic scar model or Murine mechanical distraction model.

Key biomarkers: Efficacy should be evaluated against mechanotransduction markers (YAP/TAZ, α-SMA) rather than simple collagen volume.

### 9.3 For general wound closure agents

Recommended: Splinted rodent excision serves as a high-throughput primary screen.

Gap closure: Promising candidates must be validated in porcine models due to their structural homology to human skin before clinical trials.

### 9.4 Critical perspective: beyond closure speed

Readers are cautioned against the over-interpretation of wound closure rates as the sole determinant of therapeutic efficacy. Rapid re-epithelialization does not guarantee physiological restoration and can sometimes impede proper tissue remodeling. Instead, future evaluations must prioritize qualitative endpoints, specifically histological architecture, collagen fiber alignment, and tensile strength. Ultimately, the functional regeneration of skin appendages (e.g., hair follicles and glands) represents a more clinically meaningful outcome than the mere velocity of surface coverage.

## 10 Conclusion

Our review underscores that no single animal model can fully recapitulate the complexity of human wound healing. The choice of model must be driven by the specific biological question and the intended clinical application, rather than convenience alone. We propose the following selection criteria:

- For Mechanistic Discovery: Use Murine Models (Mice). Their genetic tractability makes them ideal for elucidating specific molecular pathways (e.g., macrophage polarization, cytokine signaling). However, data must be interpreted with caution regarding contraction-mediated closure.
- For Pharmacological Screening & Ischemia: Use Rat Models. Their larger size supports complex surgical interventions (e.g., flaps, electrical burns) and repeated blood sampling for systemic toxicity/efficacy monitoring.
- For Scarring & Re-epithelialization Validation: Use Rabbit Ear or Red Duroc Pig Models. These are mandatory when the endpoint is scar quality, anti-fibrotic efficacy, or epithelial coverage, as they minimize the confounding effect of contraction (Zhu et al., 2003).
- For Pre-Clinical Regulatory Approval: Use Porcine Models. Due to their anatomical and physiological homology to humans, porcine data is often considered a prerequisite for IND (Investigational New Drug) applications (Abdullahi et al., 2014).

Animal models remain indispensable and irreplaceable *in vivo* tools for elucidating the complex, multi-cellular, and temporally dynamic mechanisms that govern skin wound healing. While *in vitro* systems and in silico simulations continue to advance, they currently lack the systemic complexity—encompassing neuro-immuno-endocrine interactions and biomechanical feedback loops—required to fully predict therapeutic outcomes in living organisms. In recent years, research interest in this field has expanded markedly, driven in large part by the increasing clinical burden of chronic and hard-to-heal wounds associated with an aging global population and the rising prevalence of metabolic syndromes such as diabetes and obesity.

In this review, we summarize the most commonly used animal models of skin wound healing —ranging from basic acute excisional defects to sophisticated pathological systems mimicking diabetic neuro-ischemic ulcers and tension-induced hypertrophic scars— and synthesize their key characteristics, strengths, and limitations. We emphasize that no single model perfectly recapitulates the human cutaneous condition; rather, each species offers a distinct window into specific aspects of the healing cascade. We further provide practical, endpoint-oriented guidance to assist investigators in selecting appropriate models for specific research objectives and translational applications. Specifically, we advocate for a strategic, hierarchical approach to model selection: leveraging the genetic tractability and cost-effectiveness of mice for high-throughput screening and mechanistic discovery (e.g., utilizing Cre-LoxP systems or optogenetics); utilizing rats and rabbits to evaluate functional endpoints such as ischemia-reperfusion injury, biofilm management, and scar remodeling (e.g., utilizing the rabbit ear hypertrophic scar model); and finally, reserving porcine models—whose skin architecture and healing kinetics most closely mirror human physiology—as the definitive “gatekeeper” for pre-clinical safety and efficacy validation prior to human trials.

Finally, we urge the scientific community to reconsider the definition of “efficacy” in preclinical trials. The prevailing fixation on “rapid wound closure” in rodent models is a poor predictor of human outcomes. Future research must pivot toward endpoints that reflect functional tissue restoration—specifically histological maturity, collagen architecture (e.g., birefringence analysis), tensile strength, and vascular perfusion (Tracy et al., 2016). Furthermore, study designs must increasingly account for “invisible” variables such as animal age, sex, and microbiome status to better simulate the complex reality of human patients. Only by rigorously addressing these limitations can we bridge the chasm between benchside discovery and bedside application. Looking forward, the integration of traditional animal modeling with emerging technologies such as single-cell spatial transcriptomics, AI-driven image analysis, and organ-on-a-chip platforms promises to further refine our understanding of wound pathology. Ultimately, the successful translation of bench-side discoveries to bedside solutions relies on the rigorous, ethically responsible (adhering to the 3Rs: Replacement, Reduction, Refinement), and scientifically rational selection of animal models that align precisely with the intended clinical context (Percie du Sert et al., 2020).

## Data Availability

The datasets presented in this study can be found in online repositories. The names of the repository/repositories and accession number(s) can be found in the article/**Supplementary Material**.

## Supplementary material

Supplementary Table 1 Detailed Search Strategy and Syntax for Electronic Databases. This table outlines the specific query strings, boolean operators, and filters applied across three major databases (PubMed, Web of Science, and Scopus) to identify relevant studies on animal models of skin wound healing. The search was restricted to articles published between January 1, 2015, and December 31, 2025, limited to the English language and animal species. Key search terms included variations of “Wound Healing,” “Skin,” and specific model descriptors such as “splint,” “silicone frame,” or “excisional wound.”

Supplementary Figure 1 Risk of bias graph.
