# Supplementary material for: Establishment and translational evaluation of animal models for skin wound healing: a systematic review
Source: Front Physiol. 2026 Apr 16;17:1800001. doi: 10.3389/fphys.2026.1800001 (PMC13128434; doi:10.3389/fphys.2026.1800001)
Supplement: Supplementary Table 1 — Detailed Search Strategy and Syntax for Electronic Databases. This table outlines the specific query strings, boolean operators, and filters applied across three major databases (PubMed, Web of Science, and Scopus) to identify relevant studies on animal models of skin wound healing. The search was restricted to articles published between January 1, 2015, and December 31, 2025, limited to the English language and animal species. Key search terms included variations of “Wound Healing,” “Skin,” and specific model descriptors such as “splint,” “silicone frame,” or “excisional wound.” [file DataSheet1.pdf]

Supplementary Table S1. Detailed Search Strategy and Syntax for Electronic Databases.

Database	Search Strategy (Query Strings)	Filters / Limits Applied
PubMed	((“Models, Animal”[Mesh] OR “Animal Experimentation”[Mesh] OR “Mice”[Mesh] OR mouse[Title/Abstract] OR mice[Title/Abstract] OR “Rats”[Mesh] OR rat[Title/Abstract] OR “Rabbits”[Mesh] OR rabbit[Title/Abstract]) AND (“Skin”[Mesh] OR “Wound Healing”[Mesh] OR “skin wound”[Title/Abstract] OR “incisional wound”[Title/Abstract] OR “excisional wound”[Title/Abstract] OR “burn wound”[Title/Abstract] OR “diabetic wound”[Title/Abstract] OR “chronic wound”[Title/Abstract] OR “hypertrophic scar”[Title/Abstract] OR fibrosis[Title/Abstract]) AND (splint[Title/Abstract] OR ring[Title/Abstract] OR “silicone frame”[Title/Abstract] OR stretch[Title/Abstract] OR tension[Title/Abstract] OR “wound creation”[Title/Abstract] OR “perioperative management”[Title/Abstract] OR “quantitative outcome”[Title/Abstract]))	Date: 2015-01-01 to 2025-12-31; Language: English; Species: Other Animals
Web of Science (Core Collection)	TS=((“Models, Animal” OR “Animal Experimentation” OR Mice OR mouse OR “Rats” OR rat OR “Rabbits” OR rabbit) AND (“Skin” OR “Wound Healing” OR “skin wound” OR “incisional wound” OR “excisional wound” OR “burn wound” OR “diabetic wound” OR “chronic wound” OR “hypertrophic scar” OR fibrosis) AND (splint OR ring OR “silicone frame” OR stretch OR tension OR “wound creation” OR “perioperative management” OR “quantitative outcome”))	Date: 2015-01-01 to 2025-12-31; Language: English; Document Type: Article
Scopus	TITLE-ABS-KEY((“Models, Animal” OR “Animal Experimentation” OR Mice OR mouse OR Rats OR rat OR Rabbits OR rabbit) AND (Skin OR “Wound Healing” OR “skin wound” OR “incisional wound” OR “excisional wound” OR “burn wound” OR “diabetic wound” OR “chronic wound” OR “hypertrophic scar” OR fibrosis) AND (splint OR ring OR “silicone frame” OR stretch OR tension OR “wound creation” OR “perioperative management” OR “quantitative outcome”))	Date: 2015-01-01 to 2025-12-31; Language: English; Document Type: Article

This table outlines the specific query strings, boolean operators, and filters applied across three major databases (PubMed, Web of Science, and Scopus) to identify relevant studies on animal models of skin wound healing. The search was restricted to articles published between January 1, 2015, and December 31, 2025, limited to the English language and animal species. Key search terms included variations of "Wound Healing," "Skin," and specific model descriptors such as "splint," "silicone frame," or "excisional wound."
