# Supplementary figures and images for: Establishment and translational evaluation of animal models for skin wound healing: a systematic review
Source: Front Physiol. 2026 Apr 16;17:1800001. doi: 10.3389/fphys.2026.1800001 (PMC13128434; doi:10.3389/fphys.2026.1800001)

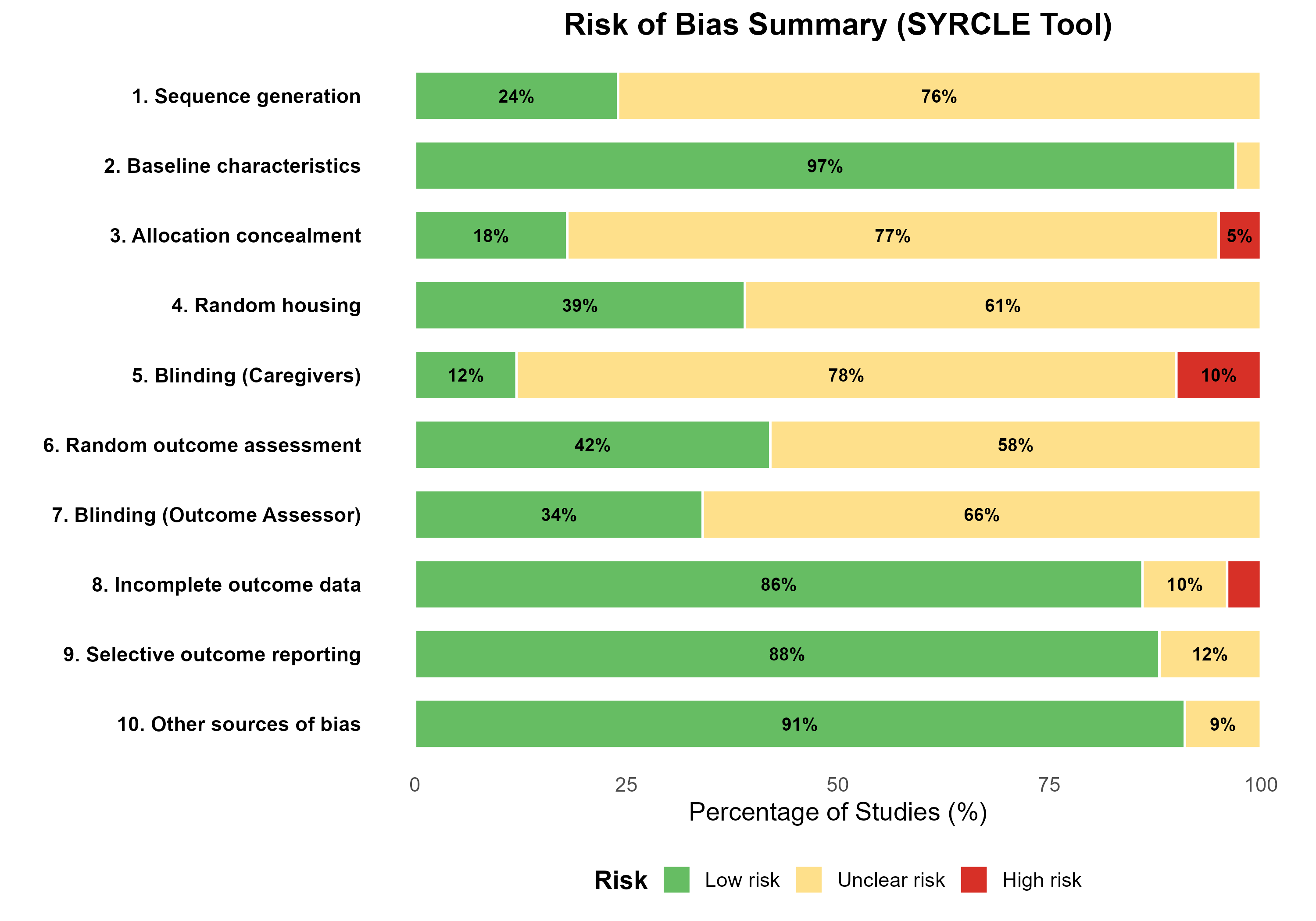

Supplement: Supplementary Figure 1 — Risk of bias graph. [file Image1.tiff]
